# A Systems Biology Approach Towards a Comprehensive Understanding of Ferroptosis

**DOI:** 10.3390/ijms252111782

**Published:** 2024-11-02

**Authors:** Mikhail Arbatskiy, Dmitriy Balandin, Ilya Akberdin, Alexey Churov

**Affiliations:** 1 Russian Clinical Research Center of Gerontology, Pirogov Russian National Research Medical University, Ministry of Healthcare of the Russian Federation, 129226 Moscow, Russia; d.balandin01@yandex.ru (D.B.); achurou@yandex.ru (A.C.); 2 Department of Computational Biology, Scientific Center for Genetics and Life Sciences, Sirius University of Science and Technology, 354340 Sochi, Russia; akberdin.ir@talantiuspeh.ru

**Keywords:** systems biology, mathematical modeling, ferroptosis, neurodegenerative diseases

## Abstract

Ferroptosis is a regulated cell death process characterized by iron ion catalysis and reactive oxygen species, leading to lipid peroxidation. This mechanism plays a crucial role in age-related diseases, including cancer and cardiovascular and neurological disorders. To better mimic iron-induced cell death, predict the effects of various elements, and identify drugs capable of regulating ferroptosis, it is essential to develop precise models of this process. Such drugs can be tested on cellular models. Systems biology offers a powerful approach to studying biological processes through modeling, which involves accumulating and analyzing comprehensive research data. Once a model is created, it allows for examining the system’s response to various stimuli. Our goal is to develop a modular framework for ferroptosis, enabling the prediction and screening of compounds with geroprotective and antiferroptotic effects. For modeling and analysis, we utilized BioUML (Biological Universal Modeling Language), which supports key standards in systems biology, modular and visual modeling, rapid simulation, parameter estimation, and a variety of numerical methods. This combination fulfills the requirements for modeling complex biological systems. The integrated modular model was validated on diverse datasets, including original experimental data. This framework encompasses essential molecular genetic processes such as the Fenton reaction, iron metabolism, lipid synthesis, and the antioxidant system. We identified structural relationships between molecular agents within each module and compared them to our proposed system for regulating the initiation and progression of ferroptosis. Our research highlights that no current models comprehensively cover all regulatory mechanisms of ferroptosis. By integrating data on ferroptosis modules into an integrated modular model, we can enhance our understanding of its mechanisms and assist in the discovery of new treatment targets for age-related diseases. A computational model of ferroptosis was developed based on a modular modeling approach and included 73 differential equations and 93 species.

## 1. Introduction

Cellular and animal models have revolutionized the study of biological processes in biology and medicine, presenting new opportunities for advancing the study of many biological processes [1,2]. The emergence of mathematical modeling and systems biology approaches has enabled the construction of accurate mathematical models that represent fundamental patterns in the subject being studied and provide a powerful tool for the prediction of the system response to both internal and external modifications [3,4].

Ferroptosis is a type of controlled cell death that occurs when there is an excess of peroxidized phospholipids (PL). Intracellular iron has been linked to this particular form of cell death, which is differentiated from other types of cell death in terms of morphology, biochemistry, and genetics [5]. In a steady state of cellular functioning known as homeostasis, ferroptosis is carefully controlled by the antioxidant system, where the crucial role of converting lipid hydroperoxides to lipid alcohols is performed by glutathione peroxidase 4 (GPX4) [6].

It is worth noting that a prevalent way to trigger ferroptosis is through the disturbance of iron metabolism and increased generation of reactive oxygen species. To break off ferroptosis, one can utilize two types of chemical compounds: antioxidants and iron ion chelators. Nonetheless, these compounds can only impact ferroptosis at the metabolic level and do not directly interact with its execution. The primary mechanisms of ferroptosis, including lipid peroxidation, iron metabolism, cellular antioxidant systems, and NADPH-dependent enzymes, have been well investigated [7]. Due to the aforementioned processes, the proportion of oxidized cell metabolites can greatly increase in comparison to the reduced forms. Due to the unpredictable nature of each process’s contribution in cellular models, it is necessary to study ferroptosis via a modeling approach to evaluate their importance and involvement in the initiation and progression of this process.

The publication by Mitchell et al. introduces a mechanistic computational model of iron metabolism in the human liver, which incorporates the key regulatory components [8]. It was constructed using established regulatory mechanisms and their corresponding kinetic properties, sourced from various publications. The model’s accuracy was confirmed by quantitatively comparing its results to existing physiological data. It can replicate a wide range of experimental findings. The kinetics and reaction of the system to oral iron supplementation were simulated and compared to a clinical trial, resulting in a model that accurately reproduced the observed time course. The model has the potential to predict the characteristics of the iron system in the liver and understand the connection between genotype and phenotype; additionally, it offers the opportunity to investigate hypothetical situations that lack experimental data, enabling effective experiment planning.

Parmar and colleagues focused on creating a mathematical framework for determining iron distribution in mice, using ferrokinetic data for calibration [9]. Afterward, it was confirmed using mouse models of iron deficiency diseases like hemochromatosis, β-thalassemia, atransferrinemia, and inflammatory anemia. To accurately account for the ferrokinetic data, the following mechanisms were necessary: (a) transferrin-mediated iron delivery to tissues; (b) the hepcidin induction of transferrin-bound iron; (c) ferroportin-dependent iron export regulated by hepcidin; and (d) the erythropoietin regulation of erythropoiesis. The application of the model was used to examine how erythropoiesis and erythrocyte half-life impact thalassemia symptoms. By analyzing the results of various transferrin treatment plans in patients with β-thalassemia, the effectiveness of the model in simulating disease intervention was proven.

A mathematical model was developed to examine the process of hemopoiesis in humans, incorporating the use of hematopoiesis-stimulating medications and iron supplements [10]. The existing human erythropoiesis model was integrated with advanced pharmacokinetic models for administering hematopoiesis-stimulating drugs, as well as a newly developed iron metabolism model that incorporates iron supplementation [11]. The ordinary differential equations were formulated by converting established biological mechanisms. A thorough examination of the model’s qualitative characteristics was conducted, taking into account various interventions including bleeding, iron deficiency, and drug treatment. The model can explain the temporal characteristics of red blood cells, reticulocytes, hemoglobin, hematocrit, erythrocytes, erythropoietin, serum iron, ferritin, transferrin, and transferrin saturation under different scenarios including under erythropoiesis stimulation drugs and iron administration to healthy volunteers or chemotherapy patients.

The primary aim of the other project was to develop a model that accurately depicts the kinetics of ferritin iron sequestration and could be integrated into more comprehensive cellular models; additionally, it can aid in comprehending the larger picture of how ferritin iron is stored in cells. The values for the model’s parameters were obtained through established kinetic and binding experiments, and its accuracy was confirmed by comparing it to separate data not utilized during its creation. The results of the modeling indicated that the level of ferritin had the most significant impact on the sequestration process, while the degree of iron saturation within the ferritin (the number of iron atoms sequestered per cell) fine-tuned the initial rates. The model is constructed with the crucial details of ferritin kinetics, includes a limited number of reactions and species, and can easily accommodate minor variations in the subunit composition. It is suggested that this can act as a foundational component in different models of iron metabolism for particular cell types [12]. This ferritin model has been utilized to analyze different components of iron binding dynamics, and the anticipated outcomes align with the actual experimental results.

The Shamsi et al. study utilized molecular docking and molecular dynamics (MD) modeling techniques to perform the structural docking of quercetin and naringenin to human ferritin in the context of diabetes mellitus [13]. Initially, the affinities of the quercetin and naringenin for the ferritin were examined, followed by a detailed investigation of their strong interactions to identify stable poses. Following a 100 ns all-atom molecular dynamic simulation, a docking procedure was conducted and subsequently analyzed using principal component analysis and free energy landscape analysis. The paper’s authors propose that quercetin and naringenin possess the potential to serve as ferritin-targeted substances in the creation of treatments for Alzheimer’s disease.

Konstorum and colleagues created a discrete, stochastic, multivariable mathematical model to explain the regulation of ferroptosis. The eleven variables included in various modules are depicted in the model. In the PL precursor pathway, there are saturated fatty acids (SFA), stearoyl CoA desaturase 1 (SCD1), monounsaturated fatty acids (MUFA), phosphatidylcholine acyltransferase (LPCAT3), arachidonic acid (AA), acyl-CoA synthetases (ACSL4), and acetylated polyunsaturated fatty acids (PUFA-CoA). In the PL peroxidation pathway, there are the labile iron pool (LIP), reactive oxygen species (ROS), lipoxygenase (ALOX15), phospholipid attached to polyunsaturated fatty acids (LH-P), lipid hydroperoxide (L-OOH), peroxyl radical (LO*), and glutathione peroxidase (GPX4). In the GPX precursor pathway, there are glutathione (GSH), glutathione disulfide (GSSG), reduced nicotinamide adenine dinucleotide phosphate (NADPH), and an oxidized form of nicotinamide adenine dinucleotide phosphate (NADP+) [14].

The variables have a range of three potential values that correspond to the low, medium, and high activity or expression levels of molecules or proteins. The predefined set of rules and asynchronous updating scheme is utilized to update the variables at every discrete time step. Five external inputs are employed in the simulation—ACSL4, SCD1, ferroportin, transferrin receptor, and p53—to analyze the impact of various signals and perturbations on the induction of ferroptosis. These inputs remain constant throughout the simulation [14].

Konstorum and colleagues conducted a system-level analysis to examine the effects of various input conditions and parameters on the sensitivity to ferroptosis. The study revealed that the susceptibility to ferroptosis is determined by the equilibrium between the pro-oxidants (ROS, lipoxygenase) and antioxidants (GPX4). Changing certain parameters can decrease the sensitivity to ferroptosis by reducing the L-OOH production. Elevated ACSL4 and reduced SCD1 levels lead to a heightened susceptibility to ferroptosis. The model’s numerical analysis revealed that increased SCD1 levels can impede the initiation of ferroptosis, even in the presence of high ACSL4 levels and low SCD1 levels. The model’s predictions were validated through an in vitro experimental system involving an ovarian cancer stem cell culture. The model effectively explains how the signaling pathways from various inputs interact to trigger ferroptosis.

The signaling networks of the most prevalent types of cell death were outlined in a research paper. According to the authors, cell death is caused by the convergence of various established cell death mechanisms. Hence, it is crucial to understand not just one but the shared regulatory factors that influence cell death and can be utilized for control [15].

Kagan and colleagues created a continuum framework for ferroptosis, centered on the biochemical processes involved in arachidonic acid metabolism and GPX4 control. The design of the system was based on 47 ordinary differential equations and separated into three distinct modules: arachidonic acid metabolism, arachidonic acid-triggered ferroptosis, and a module for regulating GPX4. The experimental data corroborated the model’s predictions [16].

Agmon and colleagues shared their findings from the molecular dynamic simulations of membranes implicated in ferroptosis-mediated cell death. The research demonstrated the impact of ferroptotic lipid components on altering the biophysical properties of membranes [17]. The model utilized a less complex membrane design compared to that found in real cells. The simplification was to propose a potential mechanism for membrane disruption, but it may not consider additional vital processes that take place in living membranes during ferroptosis damage. The gathered data revealed that ferroptotic lipids possess a distinct structure compared to conventional lipid forms, hindering their even distribution in the bilayer. When these lipids accumulate, it can result in significant disruptions to the membrane.

According to other published works, a network model of avascular ferroptosis has been suggested, utilizing experimental findings to demonstrate the connections between the ferroptotic pathway and the growth of vascular tumors [18,19]. Should the model’s performance be validated, the achieved outcomes can be utilized to strategize anticancer treatment by promoting ferroptosis, potentially resulting in a less severe disease progression and mitigating the harmful side effects of therapy.

Having reviewed the literature on modeling the individual modules of the model we presented, we concluded that, as of today, there is no modular integrative model that includes all the main causes of ferroptosis development described in the literature as modules. The aim of this work is to create the first modular model that facilitates the prediction of cellular behavior under conditions promoting ferroptosis development and to study the response of both individual modules and the entire integrative model to changes in the included parameters. The ultimate goal is to use this model to assess the regulatory potential of new compounds capable of inhibiting ferroptosis development as one of the pathogenetic links in the development of age-associated diseases. In our model, we reflected the main causes of ferroptosis development and divided them into six modules: the Fenton and Haber–Weiss reactions, iron metabolism, lipid synthesis, lipid peroxidation, the pentose phosphate pathway, and the antioxidant system. To build the model, we used the BioUML platform, which is designed for the modular modeling of complex biological systems.

All the details on the general description of each module, the representation of the module diagram using extended SBGN Process Description notation, the corresponding Antimony code for the module, as well as the reaction rates and ordinary differential equations to describe the species concentrations, the module parameters, their values, and the references to the literature from which they were extracted are presented in the Supplementary Materials.

## 2. Results

### 2.1. Model Overview

**Figure 1:**
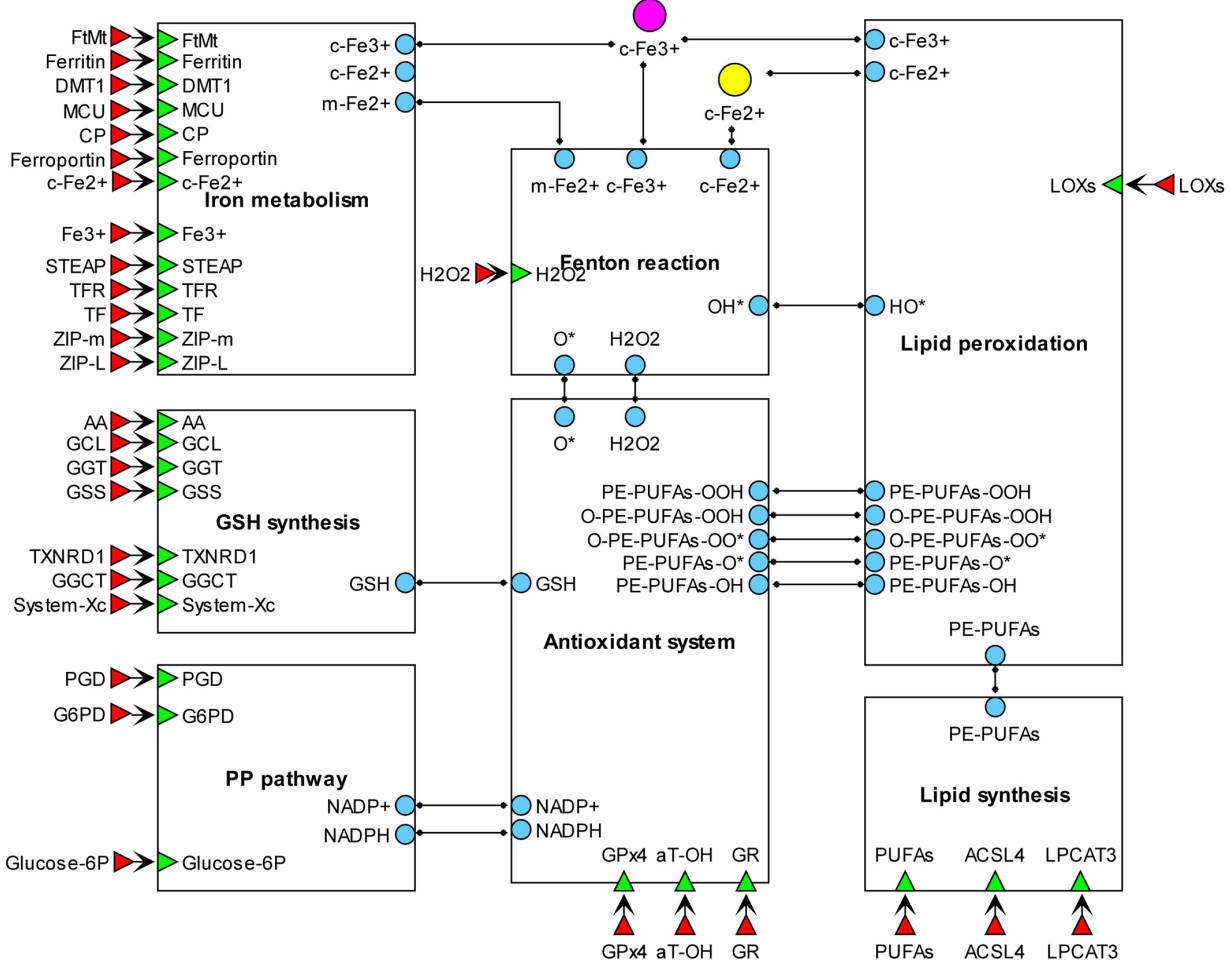
Modular diagram of the ferroptosis model.

As mentioned earlier, the process of ferroptosis occurs when iron participates in the peroxidation of polyunsaturated lipids, and this occurs within the appropriate module wherein, in addition to iron, reactive oxygen species play a role in the oxidation mechanism. The iron required for the oxidation processes and Fenton reactions that produce reactive oxygen species (ROS) originates from the iron metabolism module. The levels of proteins involved in importing and reducing the labile iron pool within the cell through ferritinophagy, along with ferritin and ferroportin, play a significant role in the development of ferroptosis (Figure 1).

To sustain the advancement of ferroptosis, it is crucial to have polyunsaturated phospholipids, the production of which utilizes the regulators of fatty acid metabolism (ACSL4) and lipid modification (LPCAT3); the sensitivity of cells to ferroptotic death is determined by their activity.

In the upcoming section, we will examine each of the model modules individually. Each module is accompanied by a detailed description of its elements, as well as information on existing models and a comparison of our modules to them.

### 2.2. Fenton’s Reaction

**Figure 2:**
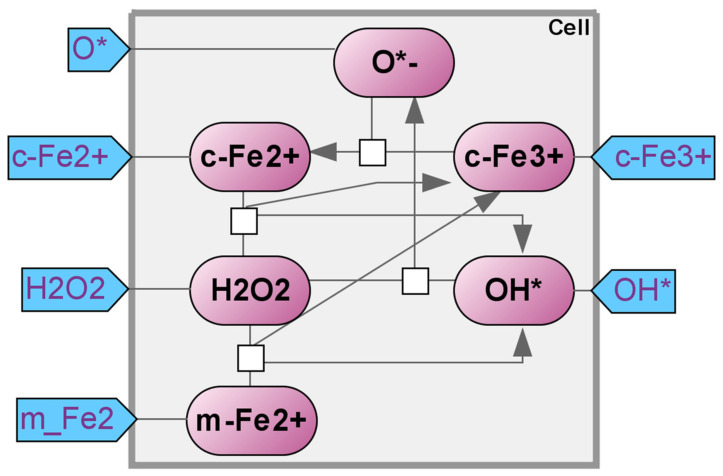
Fenton’s reaction module. c-Fe^2+^—cytoplasmic labile Fe^2+^ stock; c-Fe^3+^—cytoplasmic labile Fe^3+^ stock; m-Fe^2+^—mitochondrial labile Fe^2+^ stock; O*^−^—oxygen radical anion; H_2_O_2_—water; OH*—hydroxide radical.

Fenton’s reaction is the oxidation of the iron ion from Fe^2+^ to Fe^3+^ [20]. Typically, the hydrogen peroxide molecule breaks down into a hydroxide radical and a hydroxide anion. Through this process, the hydrogen peroxide takes in both an electron and a hydrogen proton. In the Fenton reaction, the iron ion Fe^2+^ serves as the electron donor (Figure 2).

There are currently no articles available that offer a mathematical representation of the Fenton reaction in relation to the ferroptosis process. The reaction mechanism remains consistent regardless of the medium, allowing for the utilization of calculated kinetics from individual steps in other research contexts. An article discussing the use of the Fenton reaction for processing non-biodegradable wastes highlights that the reaction can be broken down into two distinct stages, despite its intricate kinetics [21]. The first stage starts with the hydroxide radical (*OH) resulting from the reaction of the Fe^2+^ and H_2_O_2_ and lasts for about 120 min. During this phase, the utilization of the hydrogen peroxide is significant as a result of the catalytic generation of the *OH radical. Following this, there is a gradual phase as the Fe^2+^ becomes restricted due to the slow regeneration reaction rate, and the H_2_O_2_ gradually returns to its original level. Through various trials involving varying initial pH levels, doses of H_2_O_2_ and Fe^2+^, and concentrations of the initial filtrate, it was determined that the proposed model, which utilized two constants, effectively measured and guaranteed the success of the process.

A different study investigated how the Fenton reaction plays a role in the decomposition of crystalline violet [22]. The optimal conditions for decomposition were studied by examining the impact of pH, H_2_O_2_ levels, dye, and temperature on Fe^2+^ ions.

By examining the existing models, it is evident that they depict the components of the Fenton reaction. However, within the cell, the Fenton reaction is not the only source of hydroxyl radicals. The Haber–Weiss reaction, a spontaneous interaction between hydrogen peroxide and superoxide anion radical, also contributes to their production. Our Fenton reaction module now includes the Haber–Weiss reaction (see details in Supplementary Table S1, Kinetic equations “Fenton’s Reaction”).

### 2.3. Iron Metabolism

**Figure 3:**
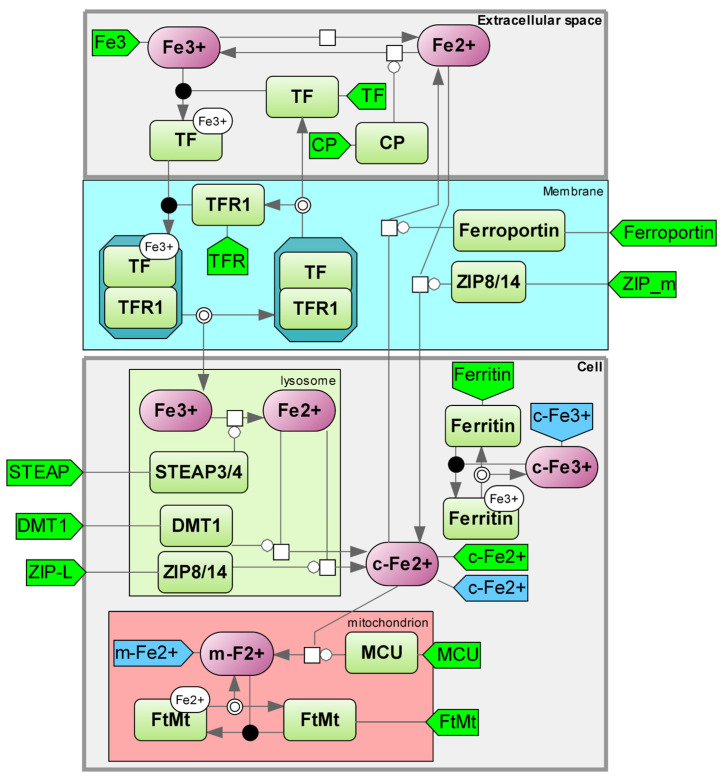
Iron metabolism module. c-Fe^2+^—cytoplasmic labile Fe^2+^; c-Fe^3+^—cytoplasmic labile Fe^3+^; m-Fe^2+^—mitochondrial labile Fe^2+^; TF—transferrin; TFR1—transferrin receptor; STEAP3/4—six-transmembrane prostate epithelial antigen 3 metalloreductase; CP—ceruloplasmin; DMT1—solute carrier family 11 member 2; ZIP8/14—solute transporter family 39 member 8/member 14; FtMt—mitochondrial ferritin; MCU—Mitochondrial calcium uniporter [23,24,25].

The internal regulation of iron levels is closely intertwined with the overall iron metabolism of the body, as the amount of iron within cells corresponds to the intake and absorption of iron from food and its transportation within the cell. The literature contains models that describe iron metabolism at both the cellular and organismal levels (Figure 3).

The first mathematical models of iron metabolism appeared as early as 1967 [26]. By utilizing a metabolic model, the iron flux in the body was determined through monitoring the kinetics of radioactive iron in the plasma. Typically, the decrease in plasma radioactivity follows a biexponential pattern, indicating the presence of a dynamic compartment in balance with the plasma. The observed uptake of radioactive iron by erythrocytes in vivo supports the accuracy of the proposed model according to kinetic analysis. The publication by Franzone et al. outlines a mathematical representation of iron metabolism [20]. The body contains the following reservoirs of iron: transferrin-bound iron in blood plasma, iron in circulating erythrocytes and their medullary precursors, iron in the cells of mucous membranes, parenchyma, and reticuloendothelial cells. The impact of erythropoietin hormone on bone marrow’s utilization of iron for hemoglobin production was considered. The resulting model consists of functional–differential equations with a delayed type. The majority of the model’s parameters were determined through radioactive experiments, while the rest were measured or given numerical values. Various treatments were found to stimulate changes in the iron metabolism. The model’s predictions were evaluated against clinical data collected in real-world settings.

The potential low-molecular-weight inhibitors of ferroptosis could be found by targeting the iron accumulation mechanisms in ferritins. The current method of utilizing iron chelators (deferiprone, deferoxamine, deferasirox) is effective, but their prolonged use is restricted. Studies have demonstrated that these substances not only inhibit the production of red blood cells but also hinder the bone marrow’s ability to generate white blood cells. Small molecules with the ability to promote iron storage in ferritin complexes and/or the stabilization of these complexes could potentially serve as effective agents in treating various diseases linked to excessive ferroptosis. Exploring the control of cellular iron levels, specifically the process of iron buildup in ferritins, presents an emerging and significant task. The inclusion of these models can enhance existing global models of ferroptosis.

The majority of developed models depict the process of iron metabolism across various organs and tissues, with only one paper concentrating on intracellular mechanisms. Our proposed structure diagram highlights the intracellular iron metabolism network and encompasses a greater number of iron metabolism components than what was shown in Mitchell’s model. Table 1, comparing the model elements, is also included (see details in Supplementary Table S2, Kinetic equations “Iron Metabolism”).

### 2.4. Lipid Synthesis

**Figure 4:**
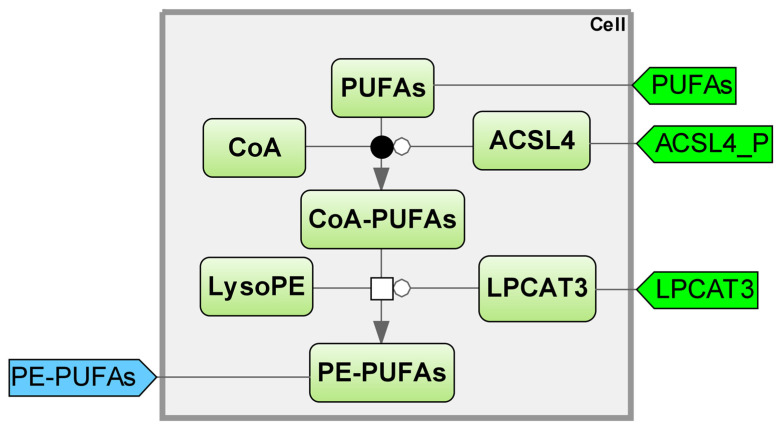
Lipid synthesis module. LPCAT3—lysophosphatidylcholine acyltransferase 3; PE–PUFAs—phosphatidylethanolamine–polyunsaturated fatty acids; CoA—coenzyme A; LysoPE—lysophosphatidylethanolamine; PUFAs—polyunsaturated fatty acids; ACSL4—acyl–CoA synthetase long-chain family member 4 [29].

The body’s lipid metabolism is closely connected to both lipid synthesis and iron metabolism, as seen in the previous module. The unique aspect of lipid metabolism is that, unlike other dietary substances, they do not enter the bloodstream directly, but instead are packaged into chylomicrons and lipoproteins before being transported to peripheral tissues [27]. The stable functioning of transport systems is crucial for providing lipids for the construction of cell walls. When constructing a model of lipid metabolism, it is crucial to take into account both intracellular processes and lipid transport to the cell [28] (Figure 4).

A mathematical model was created in one study to simulate the metabolic effects of different mixed meals in both fed and fasted states, with a specific focus on the hepatic triglyceride component [27]. The framework proposed by the model allows for the investigation of these mechanisms, which are believed to play a role in the development of hepatic steatosis. Furthermore, it serves as a foundation for examining the impact of insulin resistance on the accumulation of triglycerides in the liver and in various tissues. In another experiment, researchers examined the arteriovenous blood samples of subcutaneous adipose tissue from various locations while conducting a mixed meal test to assess the effectiveness of three current adipose tissue metabolism models and to develop a more sophisticated model of adipose tissue metabolism. The model incorporates fresh terminology that specifically considers the transformation of glucose to glyceraldehyde-3-phosphate, as well as the uptake of glycerol into adipose tissue following a meal, to better explain the observed fluxes in adipose tissue, including various delays that are biologically significant in the insulin signaling pathway [30]. The study illustrates the significant impact of insulin signaling transmission on the dynamics of adipose tissue and has been utilized to measure the effects of caloric restriction on adipose tissue metabolism.

When seeking comparable models, it may not always be feasible to locate a model that precisely aligns with our defined objective, demonstrating the existence of numerous mathematical models for the same process, depending on the specific focus of the study. In one of the studies, a mechanistic approach was utilized to develop a model for the biosynthesis of triglycerides, phospholipids, and cholesterol esters, and the assembly of very low-density lipoproteins [31]. The mathematical model serves as a fundamental structure for constructing a numerical comprehension of lipid metabolism and regulation and can be utilized to proficiently convey and assess the proposed hypothesis about lipid metabolism.

It is challenging to pinpoint the exact metabolic process of fatty acid synthesis, which plays a crucial role in ferroptosis, due to the broad nature of lipid synthesis. Therefore, incorporating a dynamic model that utilizes differential equations and reaction rate equations, which account for the synthesis of fatty acids, is essential for the overall ferroptosis model [32]. The lipid synthesis model encompasses critical stages that could potentially regulate the ferroptotic response of the cell, resulting in either enhancement or abrogation. During ferroptosis, acyl-CoA synthetase long-chain family member 4 (ACSL4) and lysophosphatidylcholine acyltransferase 3 (LPCAT3) are responsible for the production of most PUFAs and contribute to the esterification of PUFAs into phosphatidylethanolamine (PE) [33,34]. ACSL4 inhibitors regulate cell sensitivity to reduce ferroptosis [35] (see details in Supplementary Table S3, Kinetic equations “Lipid Synthesis”).

### 2.5. Lipid Peroxidation

**Figure 5:**
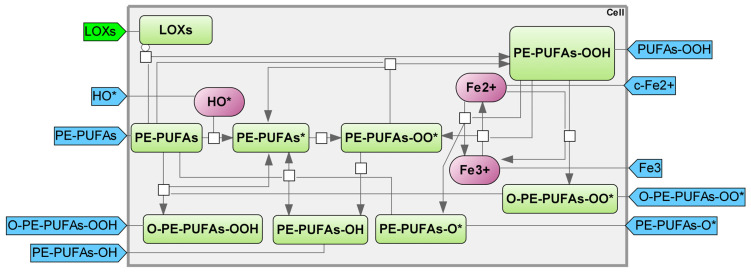
Lipid peroxidation module. PE-PUFAs—phosphatidylethanolamine–polyunsaturated fatty acids; PE-PUFAs-O-OH—PE-PUFAs hydroperoxide; PE-PUFAs-OH—PE-PUFAs alcohol; PE-PUFAs-O*—PE-PUFAs alkoxyl radical; PE-PUFAs-OO*—PE-PUFAs peroxyl radical; PE-PUFAs*—PE-PUFAs radical; O-PE-PUFAs-OO*—epoxyallylic PE-PUFAs peroxyl radicals; O-E-PUFAs-OOH—epoxyallilic PE-PUFAs hydroperoxide; LOXs—Lipoxygenases (mainly arachidonate 15–lipoxygenase and arachidonate 5–lipoxygenase) [39,40,41].

Oxidative damage often targets the unsaturated phospholipids found in cell membranes. Lipid peroxidation can occur, resulting in a degenerative process that disturbs the system’s structure and function [36]. Cell death caused by ferroptosis is a result of an overabundance of lipid peroxidation. LOXs or cellular free iron catalyze the enzymatic or non-enzymatic peroxidation of PE-PUFAs [37]. Hydroxyl radicals remove hydrogen atoms from PE-PUFA to generate a carbon-centered radical in the phospholipid. The newly formed peroxyl radical undergoes further oxidation with oxygen, leading to the production of phospholipid hydroperoxide, which plays a role in ferroptosis (Figure 5) [38].

Byczkowski and his team created a model that explains and replicates the lipid peroxidation caused by chemical substances, specifically by stimulating the production of lipid hydroperoxides and thiobarbituric acid-reactive substances in correlation with the quantity of cytochrome P450 and antioxidant system-activated glutathion [42]. With a few adjustments, the model has the potential to be used for assessing the relative intrinsic hepatotoxicity caused by lipid peroxidation in various species in vivo, such as mice, rats, and humans. It is also applicable in representing the potential pro-oxidant impacts of dietary factors such as polyunsaturated fats and antioxidants, as well as occupational and environmental toxins. Kisei thoroughly investigated the lipid peroxidation process in another study, focusing on the prevalence of RO2 radicals [43]. The author succeeded in streamlining the model into a system of two equations. Nevertheless, in practical scenarios, it is no longer feasible to examine this instance using such a straightforward set of equations (see details in Supplementary Table S4, Kinetic equations “Lipid Peroxidation”).

### 2.6. Pentose Phosphate Pathway

**Figure 6:**
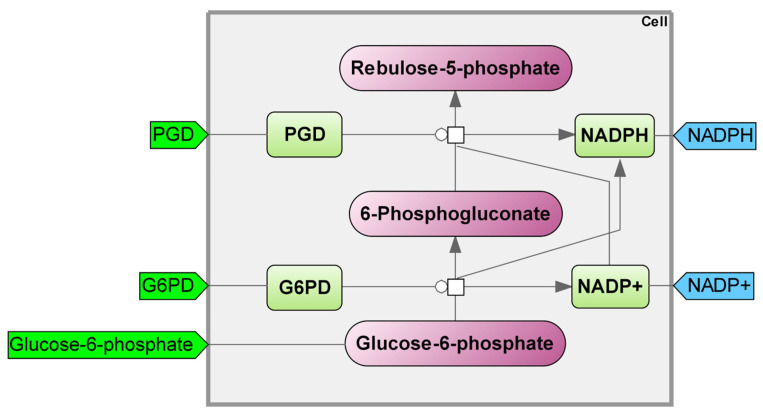
Pentose phosphate pathway module. PGD—phosphogluconate dehydrogenase; G6PD—glucose–6–phosphate dehydrogenase; NADPH—reduced nicotinamide adenine dinucleotide phosphate; NADP+—oxidized form of nicotinamide adenine dinucleotide phosphate.

The pentose phosphate pathway (PPP) serves as a substitute route for the oxidation of glucose. This mechanism furnishes cells with the NADPH coenzyme and ribose-5-phosphate. The cytosol contains all the enzymes involved in the PPP. Within this pathway, there are both oxidative and non-oxidative steps for the production of pentose from glucose (Figure 6).

Cells receive NADPH+H+ and pentoses through the oxidative process. The enzyme responsible for dehydrogenation is NADP+, which is converted to NADPH+H+. Oxidative decarboxylation produces pentoses. The oxidative step of the PPP involves the enzymes glucose-6-phosphate dehydrogenase, gluconolactone hydratase, and 6-phosphogluconate dehydrogenase.

Erythrocytes utilize NADPH+H+ to shield against reactive oxygen species. Red blood cells contain a thiol-containing tripeptide antioxidant, known as glutathione, in its reduced form, with SH groups participating in the process of transforming hydrogen peroxide into water. The reaction transforms glutathione from its reduced form to its oxidized form. The SH groups in glutathione react with H_2_O_2_ to shield cysteine residues in hemoglobin protomers from damage caused by reactive oxygen species, thereby maintaining their structural integrity and functionality. The reduced form of glutathione can be restored by utilizing NADPH+H+ as a hydrogen source, produced during the oxidative pentose phosphate reactions involved in glucose conversion, including the catalytic action of glucose-6-phosphate dehydrogenase.

One crucial aspect of this metabolic pathway is the fact that its enzymes glucose-6-phosphate dehydrogenase and 6-phosphogluconate dehydrogenase are both rate-limiting and significant producers of reduced NADPH [44], demonstrating a significantly higher efficiency compared to the alternative pathways of malic enzyme and isocitrate dehydrogenase [45]. The efficiency of antioxidant systems and maintenance of normal cellular redox status is primarily dependent on the presence of NADPH [46,47,48,49], as it is necessary for the reduction in antioxidants such as thioredoxins and glutathione.

One study examines the role of the PPP in regulating oxidative processes in cells during periods of oxidative stress. The authors suggest that the allosteric regulation of PPP enzymes and variations in the NADPH NADP ratio significantly impact the efficiency of the cell’s antioxidant system in metabolic control [50]. Our research paper proposes a model of the PPP, along with the glutathione cycle and gluconate shunt, to investigate the effects of xenobiotic-induced oxidative stress on reactive oxygen species [51]. Previous studies by other authors have examined the PPP in the liver of starved rats. Our research revealed that the oxidative stage heavily relies on glucose-6-phosphate dehydrogenase, while the non-oxidative stage is primarily driven by transketolase. ATP, ADP, inorganic phosphate, NADPH, and NADPP are the key metabolites that heavily impact the PPP [52]. Meléndez-Hevia presented a fascinating method for developing a mathematical representation of the non-oxidative phase of the PPP [53]. The model aimed to achieve the complete set of metabolites in the fewest possible steps. The study conducted by Moon and colleagues [54] investigated the role of mitochondrial NADPH as an electron donor in protecting against oxidative stress. These findings encourage the further exploration of NADPH production pathways that could potentially target transformed and normal cells, as well as cancer cells with varying lineages.

We utilized the PPP models to examine various processes. Researchers incorporated elements of PPP-related processes into the basic components of the PPP, or they eliminated some of the basic elements. The study of ferroptosis did not reveal any PPP models. Considering the distinct characteristics of ferroptosis and its significance for cellular functioning, in light of existing research, the ferroptosis module included PGD—phosphogluconate dehydrogenase; NRF2—nuclear factor erythroid 2-related factor 2; G6PD—glucose-6-phosphate dehydrogenase; NADPH—reduced nicotinamide adenine dinucleotide phosphate; NADP+—an oxidized form of nicotinamide adenine dinucleotide phosphate. Our model excludes components related to the antioxidant activity of glutathione, while other pertinent modules incorporate them (see details in Supplementary Table S5, Kinetic equations “Pentose Phosphate Pathway”).

### 2.7. Antioxidant System

The primary defense against ferroptosis is the antioxidant system, with glutathione peroxidase 4 (GPX4) being its key component. GPX4 protects cells from the peroxidation of polyunsaturated lipids by reducing hydrogen peroxide, hydroperoxides, and lipid peroxides (and their intermediates formed during oxidation) by oxidizing glutathione (GSH) to glutathione disulfide (GSSG) to form water and harmless lipid alcohols. GSSG is converted back to GSH through the action of GSSG reductase (GR) using NADPH [55]. Therefore, a redox cycle is created to regulate the GSH and GSSG ratio, which plays a crucial role in determining the cell’s redox potential. When faced with intense oxidative stress, the cell becomes unable to replenish GSSG, resulting in a depletion of GSH reserves.

**Figure 7:**
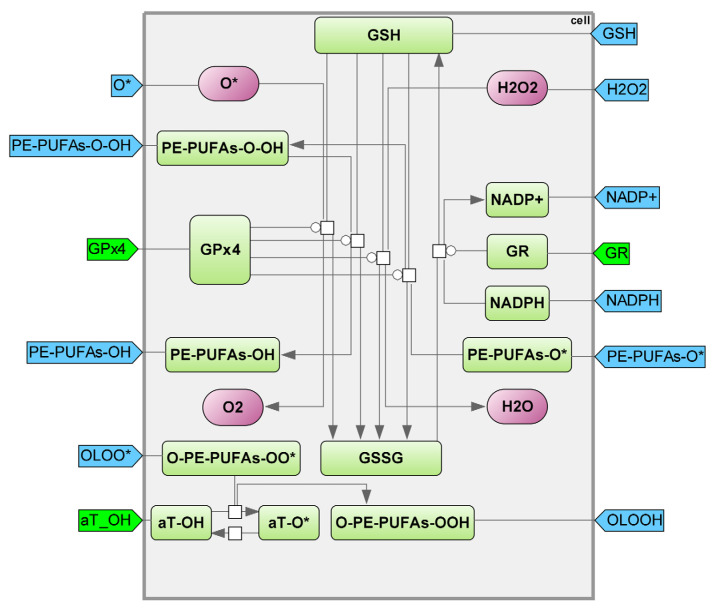
Antioxidant system module. GPX4—glutathione peroxidase 4; GR—glutathione reductase; PE-PUFAs-O-OH—PE-PUFAs hydroperoxide; PE-PUFAs-OH—PE-PUFAs alcohol; PE-PUFAs-O*—PE-PUFAs alkoxyl radical; PE-PUFAs-OO*—PE-PUFAs peroxyl radical; GSH—glutathione; GSSG—glutathione disulfide [56]; aT-OH—a-tocopherol; O-PE-PUFAs-OO*—epoxyallylic PE-PUFAs peroxyl radicals; O-PE-PUFAs-OOH—epoxyallylic PE-PUFAs hydroperoxide.

The efficiency of the antioxidant system heavily relies on the synthesis of GSH from glutamate and cysteine. The Xc system is responsible for transporting cystine into the cell in exchange for glutamate, which is crucial for GSH synthesis. Within the cell, cystine is converted to cysteine to act as a building block for GSH production [56]. The γ-glutamyl cycle facilitates the continuous production of cysteine from GSH. This crucial role is attributed to the rapid self-oxidization of cysteine to cystine in the extracellular environment, which can generate free oxygen radicals due to its high instability [57] (Figure 7).

Glutathione peroxidase 4 (GPX4) is the primary inhibitor of ferroptosis within cells, and its effectiveness is directly influenced by the concentration of glutathione (GSH), which contains cystine [58]. Consequently, blocking the XC–Cys/Glu antiporter prevents the synthesis of glutathione. As a result, the lack of GSH causes the deactivation of GPX4, leading to cell death caused by AOF-induced lipid peroxidation [59,60]. Ferroptosis causes the death of cells lacking GPX4 [61].

**Figure 8:**
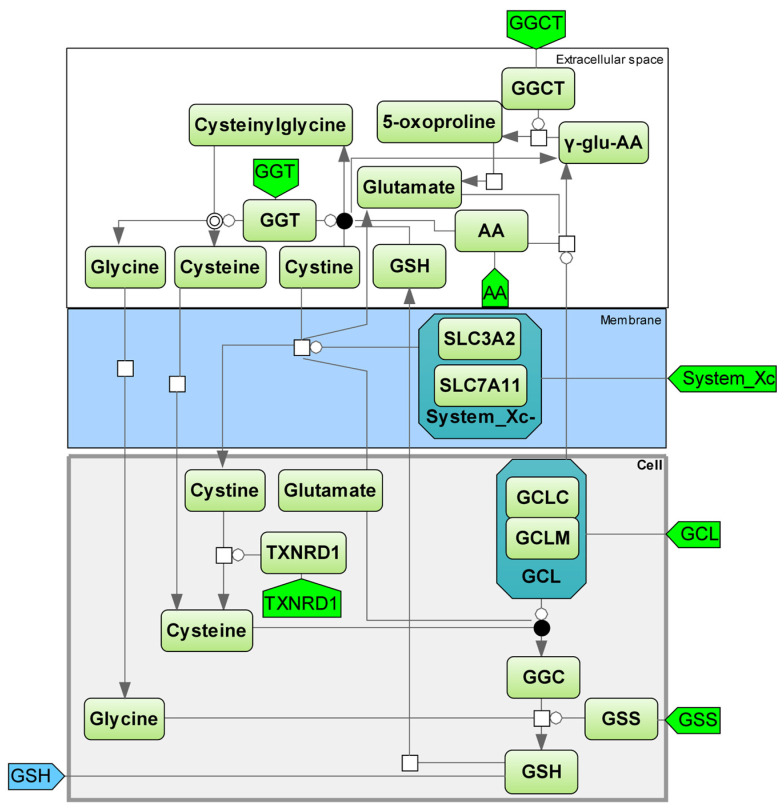
GSH synthesis module. GSH—glutathione; GGT—gamma–glutamyl transpeptidase; AA—amino acid; GGCT—γ-glutamylcyclotransferase; y-glu-AA—γ-glutamyl amino acid [55,57,62]; GCL—glutamate cysteine ligase (catalytic (GCLC) and modifier (GCLM) subunit); SLC3A2—solute carrier family 3 member 2; SLC7A11—solute carrier family 7 member 11; system_Xc^−^—cystine/glutamate exchange transporter; TXNRD1—thioredoxin reductase 1; GCL—Glutamate cysteine ligase (catalytic (GCLC) and modifier (GCLM) subunit) [63]; GGC—gamma–glutamylcysteine; GSS—glutathione synthetase [64]; GGT—gamma–glutamyl transpeptidase.

The gamma–glutamyl cycle module is responsible for the production, movement, and degradation of glutathione. Glutathione’s wide range of crucial functions can be attributed to the SH group found in its cysteine residue. The SH group causes oxidation, leading to its involvement in numerous significant processes. Many reactions involve reduced glutathione as a source of hydrogen atoms, and when two molecules are in an oxidized state, they form a dimer by creating a disulfide bond using glutathione: 2GSH → GSSG + 2H-. Glutathione reductase (GR) utilizes NADP-H to catalyze the reverse reaction: GSSG + NADP-H + H2 → 2GSH + NADP-H2 glutathione reductase (Figure 8) (see details in Supplementary Tables S6 and S7, Kinetic equations “Antioxidant System”, “GSH synthesis”).

### 2.8. Information on Available Models

The literature does not contain any works that discuss the model of the gamma–glutamyl cycle about the ferroptosis process. One of the studies included the incorporation of glutathione metabolism, one-carbon fragment metabolism, sulfur-containing amino acid pathways, and the production, transportation, and degradation of glutathione to construct a mathematical framework. A model was developed by leveraging the known traits of enzymes in the gamma–glutamyl cycle and their regulatory mechanisms in the face of oxidative stress. Were extensively studied the half-life of glutathione, the control of its production, and its responsiveness to variations in amino acid consumption. Researchers utilized the model to simulate the metabolic profile in individuals with Down syndrome and autism and subsequently compared the modeling outcomes with clinical data [65]. In a separate publication, the current gamma–glutamyl cycle model was simplified from 60 to 24 reactions by eliminating the folic acid metabolic pathway and was subsequently expanded to 41 reactions through the incorporation of a drug neutralization mechanism. The model focused primarily on the levels of glutathione and did not factor in any alterations in the proportion of its oxidized and reduced forms. The model was employed to simulate the changes observed when paracetamol (acetaminophen) was given to THLE cells derived from genetically modified human liver cells expressing human cytochrome P452E1 [66]. Kavdia’s study presents a dynamic model of the antioxidant system that thoroughly examines the dispersion and levels of NO, O^2−^, and ONOO^−^ during both normal and pathological states [67]. Research has resulted in the creation of the initial mathematical framework for the glutathione antioxidant system present in the outer layer of the retina, incorporating all the essential elements involved in the generation of reactive oxygen species, the creation of glutathione, its oxidation during the neutralization of reactive oxygen species, and the subsequent reduction in NADPH [68]. The model was calibrated and validated using experimental data.

Our proposed antioxidant system model places significant emphasis on the crucial role of interaction with oxidized lipids in countering ferroptosis. Additionally, the model incorporates a-tocopherol alongside glutathione, as it aids in the transformation of peroxidized radical forms of lipids into non-radical substances, effectively halting the chain growth reaction; additionally, the cell is safeguarded against increased levels of peroxidized lipids [69]. In the process of the gamma–glutamine cycle, the researchers considered the non-canonical function of GCL, which is activated when there is a shortage of cysteine. This deficiency has been linked to the occurrence of ferroptotic cell death. In environments like these, GCL replaces cysteine while adding other amino acids to glutamate, effectively safeguarding the cell against glutamate build-up and the possibility of ferroptosis [63]. Glutamate induces the build-up of AOF during cystine deprivation, although the precise mechanism remains unclear [5].

### 2.9. Model Validation

**Figure 9:**
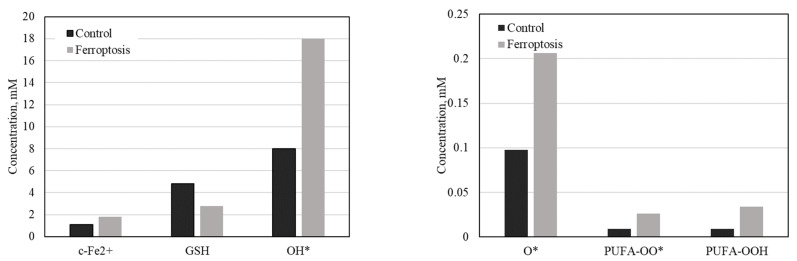
Modeling results under normal conditions and during ferroptosis. c-Fe^2+^: The level of ferrous iron (Fe^2+^) slightly increases during ferroptosis. This aligns with the understanding that ferroptosis is associated with iron accumulation, which facilitates lipid peroxidation and contributes to oxidative damage, leading to cell death [8,70]. GSH (glutathione): There is a decrease in glutathione levels during ferroptosis. Glutathione is crucial in defending cells against oxidative stress, and its depletion leaves cells more susceptible to damage, thereby promoting ferroptosis [71,72]. OH· (hydroxyl radical): There is a significant increase in hydroxyl radicals. These radicals indicate heightened oxidative processes, which play a key role in the damage to cellular components during ferroptosis [73,74]. O_2_^−^—superoxide anion: The level of superoxide anion also rises, suggesting an increase in oxidative stress and reactive oxygen species (ROS) production, further driving the cell towards ferroptotic death [75,76]. PUFA-OO· and PUFA-OOH (peroxides of polyunsaturated fatty acids): An increase in these markers points to enhanced lipid peroxidation, which is a central mechanism in ferroptosis. Lipid peroxides accumulate and contribute to membrane damage and cell death [77].

To validate the model’s functionality, two sets of external system parameters were selected based on the literature sources. One corresponds to normal physiology, while the other induces ferroptosis. The modeling results were compared with known experimental data for six parameters, which we believe most comprehensively describe ferroptosis: c-Fe^2+^, GSH, OH*, O*, PUFA-OO*, and PUFA-OOH. As can be observed, the levels of these parameters under both normal conditions and during ferroptosis development align with the data obtained from the experimental models (Figure 9).

In conclusion, the observed changes in these parameters are consistent with existing knowledge: ferroptosis is characterized by iron-induced lipid peroxidation, the depletion of antioxidants like glutathione, and increased oxidative stress, ultimately leading to cell death. These findings help explain the biochemical underpinnings of ferroptosis and its contribution to pathology in diseases associated with oxidative damage.

### 2.10. Model Constraints and Further Ways for Development

Our model provides proof of the concept of ferroptosis. The modular approach used in this study has demonstrated a methodological basis for the qualitative and quantitative development of the complex model. The analysis completed during this study allows us to refine the roadmap for further model improvements, linking this in silico version to in vivo ferroptosis in cell culture. The roadmap includes an improvement of our model by refining parameters, obtaining proprietary data from human ferroptosis cell models, and validating them in cell cultures that replicate the ferroptosis process.

## 3. Discussion

Our modular model incorporates the fundamental molecular genetic mechanisms involved in the execution of ferroptosis: the Fenton reaction, iron metabolism, lipid synthesis, lipid peroxidation, the pentose phosphate pathway, and the antioxidant system represented by the gamma–glutamyl cycle and GPX4 system.

To determine the components to incorporate into the structural model, we examined studies that focused on modeling the individual modules of ferroptosis. This was followed by a comparison between the elements included in the existing models and those proposed in our model.

After analyzing the current models of the Fenton reaction, it is clear that they accurately represent the components. However, it should be noted that hydroxyl radicals are not solely produced through the Fenton reaction within the cell. The Haber–Weiss reaction, which is a spontaneous interaction between hydrogen peroxide and superoxide anion radical, also plays a role in their generation. Therefore, we have updated our Fenton reaction module to incorporate the Haber–Weiss reaction.

The majority of developed models depict the process of iron metabolism across various organs and tissues, with only one paper concentrating on intracellular mechanisms. Our proposed structure diagram highlights the intracellular iron metabolism network and encompasses a greater number of iron metabolism components than what was shown in Mitchell’s model.

When seeking comparable lipid synthesis models, it may not always be feasible to locate a model that precisely aligns with our defined objective, demonstrating the existence of numerous mathematical models for the same process, depending on the specific focus of the study. It is challenging to pinpoint the exact metabolic process of fatty acid synthesis, which plays a crucial role in ferroptosis, due to the broad nature of lipid synthesis. Therefore, incorporating a dynamic model that utilizes differential equations and reaction rate equations, which account for the synthesis of fatty acids, is essential for the overall ferroptosis model.

To date, researchers have succeeded in streamlining the model of lipid peroxidation into a system of two equations. Nevertheless, in practical scenarios, it is no longer feasible to examine this instance using such a straightforward set of equations.

Our pentose phosphate pathway model excludes components related to the antioxidant activity of glutathione, while other pertinent modules incorporate them.

Our proposed antioxidant system model places a significant emphasis on the crucial role of interactions with oxidized lipids in countering ferroptosis.

We chose to incorporate elements into the proposed model due to its significant role in ferroptosis. The authors’ expertise in both systems biology and experimental research on ferroptosis confirms the significance of the selected elements through their experience and the structural model created is of great value due to its ability to inhibit ferroptosis in cellular models.

## 4. Material and Methods

**Figure 10:**
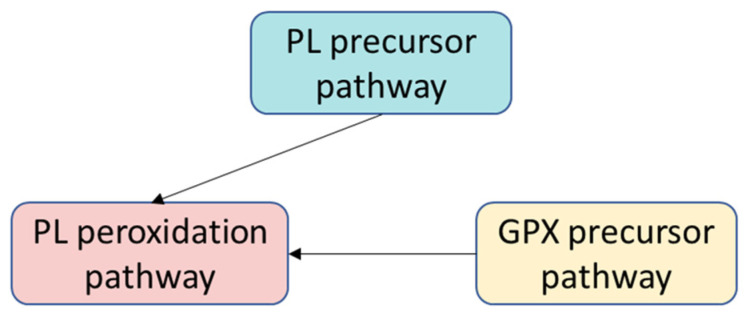
General scheme of the ferroptosis model (adapted from [14]).

To build a ferroptosis model, one must gather and assess data on its components, how they interact, its reaction rates, and its kinetic principles. We gathered data on current models about the individual components of ferroptosis. *Fenton’s reaction:* [20]. *Iron metabolism*: Najean et al. investigated various kinetic models of iron metabolism under normal conditions [26]. Colli Franzone et al. presented a mathematical model of iron metabolism, incorporating key iron pools and erythropoietin’s control on bone marrow [78]. Mitchell et al. developed a mechanistic computational model of human liver iron metabolism, providing insights into iron regulation and simulating liver iron overload dynamics, validated against clinical data [8]. Parmar et al. created a mathematical model of iron distribution in mice to better understand and simulate iron-related diseases and treatment outcomes [9]. Schirm et al. developed a biomathematical model to simulate EPO and iron medication effects in humans, aiming to optimize anemia therapy and manage iron overload risks [10]. Masison et al. proposed efficient computational models using smaller sub-models, like one for ferritin iron sequestration, to represent cellular iron metabolism dynamics and facilitate their integration into larger cell models [12]. Shamsi et al. presented an efficient computational model for cellular iron metabolism by focusing on ferritin iron sequestration kinetics, enabling their integration into larger models and enhancing the understanding of iron dynamics [13]. *Lipid synthesis*: Pratt et al. developed and validated a mathematical model to simulate metabolic responses to mixed meals, focusing on hepatic triglycerides [27]. O’Donovan et al. refined computational models of adipose tissue metabolism to better describe NEFA dynamics and insulin sensitivity [28]. Shorten et al. developed a mathematical model to predict how changes in substrate availability affect VLDL lipid composition and plasma lipoprotein lipid FA composition in the liver [31]. Kuate et al. explored dynamic modeling using ordinary differential equations to understand the complex processes of fatty acid synthesis and modification [32]. Lee et al. examined the relationship between lipid metabolic pathways, lipid peroxidation, and ferroptosis, highlighting potential treatment strategies for ferroptosis-related diseases [34]. *Lipid peroxidation*: Girotti et al. reviewed the mechanisms and measurement of unsaturated lipid peroxidation, focusing on free-radical and non-radical processes influenced by metal ions, reducing agents, and antioxidants [36]. Wu et al. characterized ferroptosis as a regulated cell death driven by iron-induced lipid peroxidation, differing from other cell death types and linked to degenerative diseases, with a potential for targeted cancer therapy and involving significant mitochondrial regulation [37]. Yu et al. identified ferroptosis as an iron-dependent cell death distinct from apoptosis and others, linked to lipid peroxidation and iron metabolism, playing a crucial role in oxidative stress, inflammation, and cardiovascular diseases, suggesting potential therapeutic targeting [38]. Byczkowski et al. developed a biologically based pharmacodynamic model to simulate chemically induced lipid peroxidation in mouse liver slices, accurately predicting experimental results and adjusting for specific biochemical parameters [42]. Volkov et al. developed a scheme for lipid peroxidation in membranes using differential equations, showing that including antioxidant fluxes can lead to oscillatory and hysteresis behaviors [43]. *The pentose phosphate pathway*: Ulusu et al. and Eshchenko et al. assessed the effects of stobadine (ST) and vitamin E on oxidative stress in diabetic rats, showing that their combination can help mitigate enzyme activity alterations and oxidative damage more effectively than antioxidants alone [44,45]. Dringen et al. confirmed the crucial role of glutathione in brain cell defense against oxidative stress, with astrocytes significantly contributing to its metabolism and protection mechanisms, vital for mitigating neurological disorders [46]. Fernandez-Fernandez et al. noticed that neuronal survival in the brain depends on antioxidant protection from astrocytes and an intrinsic glucose metabolism pathway linking to antioxidant defense, highlighting the importance of neuron–astrocyte cooperation for potential therapeutic targets in neurodegenerative disorders [47]. Chen et al. hypothesized that HIF-1alpha siRNA can protect the brain from ischemic damage by reducing apoptosis-related proteins and improving outcomes in a rat model of cerebral ischemia [48]. Deponte et al. revealed that glutathione-dependent enzymes exhibit diverse structures and mechanisms crucial for metabolism and redox regulation, yet many fundamental properties and mechanisms remain poorly understood, impacting drug development [49]. Hurbain et al. reported that cells adapt to oxidative stress by regulating glycolysis and the PPP to enhance NADPH recycling, with a model revealing the complex enzyme regulation promoting detoxification and homeostasis [50]. Schittenhelm et al. simulated how cells respond to xenobiotic-induced oxidative stress, highlighting the role of enzyme regulation and the pentose phosphate pathway in mitigating ROS damage [51]. Sabate et al. simulated the pentose phosphate pathway in fasted rat liver, highlighting that fluxes are mainly regulated by D-glucose-6-phosphate dehydrogenase and transketolase, with significant influence from ATP, ADP, Pi, NADPH, and NADP+ [52]. Meléndez-Hevia et al. used a mathematical optimization game to show that the structure of the pentose phosphate cycle in cells, aimed at converting pentoses into hexoses, reflects the simplest enzyme mechanisms with the least steps, supporting the potential evolutionary role of simplicity in metabolic pathways [53]. Moon et al. reported that mitochondrial NADPH metabolism, crucial for protection against oxidative stress, was assessed using iNap sensors, isotopic tracers, and modeling, revealing its dynamics and relationship with oxidative stress and metabolic pathways [54]. The antioxidant system, represented by *gamma–glutamyl cycle*: Lu et al. reported that glutathione (GSH), a crucial antioxidant and modulator of various cellular processes, is synthesized in a regulated manner, and its dysregulation is linked to numerous pathological conditions, highlighting the importance of understanding its regulatory mechanisms for potential therapeutic advancements [79]. Lewerenz et al. declared that the system xc^−^ transports cystine and glutamate, playing a vital role in the oxidative stress response, neurotransmitter regulation, and cancer biology, with implications for neuroprotection and the immune response [56]. Meister et al. reported that glutathione (GSH) is a crucial antioxidant present in high levels across various organisms, playing a vital role in cellular protection, detoxification, and metabolic processes, with implications for therapeutic strategies in disease treatment [57]. The *GPX4 system*: [59,60,61] (Figure 10). Our proposed element sets differ significantly from all these models, as they were not considered in the context of ferroptosis. In the results section of this paper, you will find comprehensive descriptions and comparisons of the models. Additionally, it was crucial to analyze the established models of ferroptosis to determine the key components identified by other authors as noteworthy. It should be mentioned that a few papers have detailed models of ferroptosis. Allow us to provide a more detailed description of them.

### 4.1. Structural Model Developing

By applying a modular approach and visual modeling from the original BioUML system, a comprehensive dynamic model of ferroptosis was constructed based on the reconstructed diagram [80]. The composite model of ferroptosis is founded on the inclusion of modules that describe the primary subsystems—iron metabolism, the pentose phosphate pathway, synthesis and lipid peroxidation, the gamma–glutamyl system, and the antioxidant system—taking into account the subsystem determining the level of antioxidant factor GPX4.

**Figure 11:**
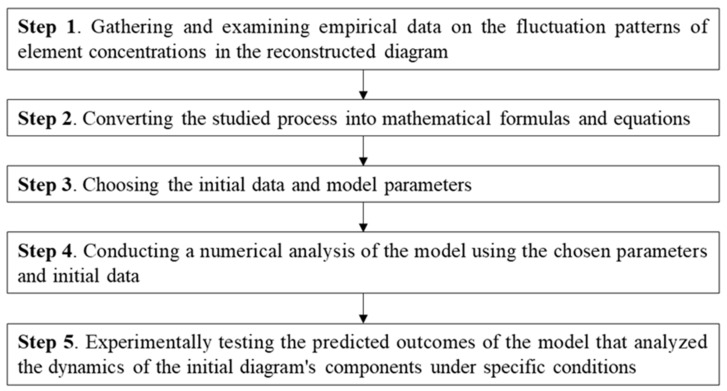
Structural model development process.

The initial step involves gathering and examining empirical data on the fluctuation patterns of element concentrations in the reconstructed diagram, under varying conditions such as ferroptosis and normal states, as well as in different age-related illnesses (Figure 11). Biologically oriented mathematical models must rely on proven experimental evidence. If there is a lack of experimental data, the model can be simplified through the use of assumptions during in silico experiments. One approach to simplifying model calculations is to treat cells and organelles as individual compartments, such as cubic cells. As a result, calculating the volumes and concentrations of substances within them becomes simpler. The use of the law of acting masses for modeling chemical kinetics requires the assumption of homogeneity within the studied cellular or subcellular system, which may not always reflect the in vivo process.

The next step in the in silico experiment involves converting the studied process into mathematical formulas and equations. These findings incorporate the fundamental principles of physics, such as the principle of mass conservation. Mathematical biology has established guidelines for representing biological phenomena in models, some of which will be explored in the following sections. Variables and parameters are essential elements in a mathematical model. In an experiment, the dynamics of substances such as transcription factors, enzymes, and metabolites are studied. These substances are known as variables. To perform calculations in the model, it is imperative to input initial data, which consists of values for the variables at the starting point of the model. The parameters in question include the rate constants of reaction, affinity, and inhibition (activation), as well as Hill’s constant, which are used to describe the cooperative effect in regulatory interactions. In this mathematical model, researchers are not concerned with the dynamics of substances, but rather with their stationary values, which can be either dimensional or dimensionless.

The third stage of constructing and analyzing the model involves choosing the initial data and model parameters. The success of this crucial step heavily relies on the biologist’s provision of necessary data for the parameter estimation. Frequently, it is challenging to empirically determine the parameters utilized in the model, yet there are several solutions to this predicament: the simplification (reduction) of the model, reducing the number of parameters as a result; the estimation of the parameter values based on indirect data (to address this issue, researchers often develop supplementary mathematical models for estimating the required parameters); determining physiologically and biochemically appropriate boundaries for the parameter values, and choosing them for the numerical analysis of the mathematical model (model fitting). The model’s uniqueness at various parameter sets becomes an issue when the number of unknown parameters is significant. Typically, in models that have a high number of unknown parameters, it is feasible to identify several parameter sets that result in a qualitatively similar model solution.

The fourth step involves conducting a numerical analysis of the model using the chosen parameters and initial data. The in silico experiment examines either the steady-state solution of the model or the temporal variations in variable values, depending on the specific problem at hand. We will outline the primary techniques for the model analysis below. If some of the parameters of the model remain unknown, it is imperative to conduct an estimation process (model fitting). The process involves repeatedly computing the model using various parameter values, with the unknown parameter being changed within predetermined boundaries. The most optimal parameters for approximating the experimental data are selected as the standard. We conduct the remaining model computations using these standard parameters. Occasionally, researchers may opt for multiple parameter sets and examine the model’s dynamics using each set separately. It is common practice to vary the parameters and analyze the model to investigate it. We utilized a model with a predetermined set of parameters as the foundation, wherein certain parameters are systematically adjusted in each calculation. A typical in silico experiment emulates the actual changes that occur in living organisms. The study findings comprise an evaluation of the system’s stability and the varying dynamics of its variable values and establishing the possible parameter ranges in which the system being studied can (or cannot) operate in a stable state. Understanding the outcomes of these computations is a crucial aspect of the in silico trial. The aim of numerically analyzing a model through the manipulation of initial data are to simulate real-life changes in a computational environment. In addition, certain in silico experiments employ advanced techniques for examining mathematical models, such as the parameter continuation method and model bifurcation analysis [81,82].

The last step involves experimentally testing the predicted outcomes of the model that analyzed the dynamics of the initial diagram’s components under specific conditions. If the developed model’s predictions do not align with experimental results in terms of quality and/or quantity, a new set of virtual experiments is carried out, beginning with adjustments to the structure and/or parameters and the initial conditions of the initial model.

### 4.2. BioUML Platform

We utilized the BioUML software (2023.1), which is specifically designed for modeling and analyzing intricate biological systems, to construct a formal graphical depiction of the components and mechanisms involved in ferroptosis [83]. Java is the coding language used to create the open-source platform, which includes a comprehensive set of tools for modeling complex systems: visual modeling, modular modeling, various mathematical formalisms and methods of model analysis, and support for SBML, CellML and SBGN notation [84].

### 4.3. Visual Modeling

Graphical representation is utilized in visual modeling to depict mathematical models through diagrams, where each element represents a mathematical object (the reaction and its participants, conditions, equations, etc.); it includes a group of modifiable factors, such as initial variable values and reaction rate equations [85]. This method enables a streamlined approach to constructing a model of a complicated biological system. The BioUML software generates efficient program code for performing model calculations using the model’s graphical representation and associated parameters.

Visual modeling relies heavily on graphical notation, which serves as the foundation for automatic code generation. The augmented notation SBGN [86], used in BioUML, integrates mathematical components from SBML [87] for modeling purposes. The system undergoes immediate changes in its state based on the conditions, using methods such as algebraic, differential, or stochastic equations [3].

### 4.4. Modular Modeling

When constructing a detailed model of a biological process, challenges are bound to arise when incorporating new components and monitoring their modifications. The situation becomes more pressing when it becomes necessary to modify or reconstruct an existing model.

This paper suggests the modular modeling of biological systems as a potential solution to these issues, a method that has seen increased interest in recent years [30,88]. Modular modeling involves breaking down a system into distinct interconnected parts known as subsystems or modules. With this approach, one can individually include and modify each element, personalize it, and examine its properties.

BioUML has designed a unique type of diagrams, called module diagrams, to incorporate this approach. These diagrams include variables, also known as ports, which facilitate the connection between modules and establish the module interface [3]. The interface consists of three types of ports: input ports, which are calculated externally; output ports, which are calculated within the module; and contacts, which can be altered both inside and outside the module (Table 2).

## 5. Conclusions

To create a model, it is necessary to have extensive knowledge regarding the physicochemical properties of the elements being studied, the kinetic laws governing the reaction processes, and the relevance of the accessible components for the examined procedure. The differential equations that describe the biological processes being studied incorporate all the possible values for the different parameters. To construct a model of the process being studied, researchers can refer to the existing literature on the function of each element or examine pre-existing process models. When examining this scenario, a crucial measure is to contrast the elements found in the current models with those to be included in the new model. The collection of components may vary greatly, yet this does not imply that one model accurately explains the process while the other does not. As there are numerous components within each model, each one has a distinct function in the overall execution of the process. When examining particular phenomena, only the essential elements relevant to the process are chosen from the entire set of elements. Additionally, it is imperative to consider the emergence of updated and more detailed information regarding the behavior of distinct elements or the progression of particular reactions, which arise due to the implementation of novel research techniques. Incorporating this technique can greatly enhance the precision of virtual process replication and improve the predictive power of the constructed model.

## Figures and Tables

**Table 1:** Comparison of our model with the model of Mitchell et al [8]. A “+” indicates the presence of the element, while a “−“ signifies its absence.

Model Element	Mitchell’s Model	Our Model	Model Element	Mitchell’s Model	Our Model
TF (transferrin)	+	+	FTMT (mitochondrial ferritin)	–	+
FT (ferritin)	+	+	ZIP8 (solute carrier family 39 member 8)	–	+
FPN1(ferroportin)	+	+	ZIP14 (solute carrier family 39 member 14)	–	+
TFR1 (transferrin receptor 1)	+	+	SLC40A1 (solute carrier family 40 member 1 (ferroportin))	–	+
TFR2 (transferrin receptor 1)	+	–	CP (ceruloplasmin)	–	+
HAMP (hepcidin)	+	–	DMT1 (SLC11A2) (solute carrier family 11 member 2)	–	+
HFE (human haemochromatosis protein)	+	–	FTH1 (ferritin heavy chain 1)	–	+
HO–1 (haeme oxygenase 1)	+	–	MCU (mitochondrial calcium uniporter)	–	+
IRP (iron response protein)	+	–	STEAP3 (six-transmembrane epithelial antigen of prostate 3 metalloreductase)	–	+
Heme	+	–			

**Table 2:** Designation of elements of the modular diagram.

Element	Designation	Description
Module	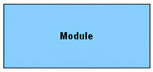	A module contains a submodel (diagram) and acts as an element of a complex modular system.
Port (input)	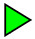	The port that inputs information into the module.
Port (contact)	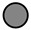	A port that allows you to edit a variable both inside and outside the module.
Port (output)	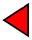	A port that transfers information from the module.
Non-directional connection	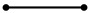	Provides communication between contact desks.
Directional connection	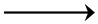	Provides communication between input and output ports.
Bus	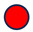	An auxiliary diagram element designed to reduce the number of overlapping connections in a modular diagram.

## Data Availability

Data are available on request.

## Supplementary Materials

The following supporting information can be downloaded at: https://www.mdpi.com/article/10.3390/ijms252111782/s1. References [89,90,91,92,93,94,95,96,97,98,99,100,101,102,103,104,105,106,107,108,109,110,111,112,113,114,115,116,117,118,119,120,121,122,123,124,125,126,127] are cited in Supplementary Materials.
