# Supplementary material for: A Systems Biology Approach Towards a Comprehensive Understanding of Ferroptosis
Source: Int J Mol Sci. 2024 Nov 2;25(21):11782. doi: 10.3390/ijms252111782 (PMC11546516; doi:10.3390/ijms252111782)
Supplement: Supplementary file 1 [file ijms-25-11782-s001.zip › Kinetic equations/Antioxidant System.html]

Differential equation system  
  

|  |  |  |  |
| --- | --- | --- | --- |
| **1** |  | time [$cell.GSH]                 2 K1 [$cell.GSH] [$cell.GPx4] [$cell.PLOOH]       K2 [$cell.GSSH] [$cell.GR] [$cell.NADPH]       K1 [$cell.GPx4] [$cell.GSH] [$cell.PE\_PUFAs\_O\_]       K4 [$cell.GPx4] [$cell.GSH] [$cell.H2O2]       [$cell.GPx4] K5 [$cell.GSH] [$cell.O\_R] |  |
| **2** |  | time [$cell.GSSH]               K1 [$cell.GSH] [$cell.GPx4] [$cell.PLOOH]       K2 [$cell.GSSH] [$cell.GR] [$cell.NADPH]       K4 [$cell.GPx4] [$cell.GSH] [$cell.H2O2]       [$cell.GPx4] K5 [$cell.GSH] [$cell.O\_R]       K1 [$cell.GPx4] [$cell.GSH] [$cell.PE\_PUFAs\_O\_] |  |
| **3** |  | time [$cell.H2O]       K4 [$cell.GPx4] [$cell.GSH] [$cell.H2O2] |  |
| **4** |  | time [$cell.H2O2]       K4 [$cell.GPx4] [$cell.GSH] [$cell.H2O2] |  |
| **5** |  | time [$cell.NADPH]       K2 [$cell.GSSH] [$cell.GR] [$cell.NADPH] |  |
| **6** |  | time [$cell.NADP\_p]       K2 [$cell.GSSH] [$cell.GR] [$cell.NADPH] |  |
| **7** |  | time [$cell.O2]       [$cell.GPx4] K5 [$cell.GSH] [$cell.O\_R] |  |
| **8** |  | time [$cell.OLOOH]     K9 [$cell.aT\_OH] [$cell.OLOOR] |  |
| **9** |  | time [$cell.OLOOR]     K9 [$cell.aT\_OH] [$cell.OLOOR] |  |
| **10** |  | time [$cell.O\_R]       [$cell.GPx4] K5 [$cell.GSH] [$cell.O\_R] |  |
| **11** |  | time [$cell.PE\_PUFAs\_O\_]       K1 [$cell.GPx4] [$cell.GSH] [$cell.PE\_PUFAs\_O\_] |  |
| **12** |  | time [$cell.PLOH]       K1 [$cell.GSH] [$cell.GPx4] [$cell.PLOOH] |  |
| **13** |  | time [$cell.PLOOH]         K1 [$cell.GPx4] [$cell.GSH] [$cell.PE\_PUFAs\_O\_]       K1 [$cell.GSH] [$cell.GPx4] [$cell.PLOOH] |  |
| **14** |  | time [$cell.aT\_OH]     K6 [$cell.aT\_OR]     K9 [$cell.aT\_OH] [$cell.OLOOR] |  |
| **15** |  | time [$cell.aT\_OR]     K6 [$cell.aT\_OR]     K9 [$cell.aT\_OH] [$cell.OLOOR] |  |

  
  
