# Supplementary material for: A Systems Biology Approach Towards a Comprehensive Understanding of Ferroptosis
Source: Int J Mol Sci. 2024 Nov 2;25(21):11782. doi: 10.3390/ijms252111782 (PMC11546516; doi:10.3390/ijms252111782)
Supplement: Supplementary file 1 [file ijms-25-11782-s001.zip › Kinetic equations/Fenton’s Reaction.html]

Differential equation system  
  

|  |  |  |  |
| --- | --- | --- | --- |
| **1** |  | time [$Cell.Fe3]         K1 [$Cell.H2O2] [$Cell.c\_LIP]     K1 [$Cell.H2O2] [$Cell.m\_Fe2]     K2 [$Cell.O\_R] [$Cell.Fe3] |  |
| **2** |  | time [$Cell.H2O2]         K1 [$Cell.H2O2] [$Cell.c\_LIP]     K1 [$Cell.H2O2] [$Cell.m\_Fe2]     K4 [$Cell.OHr] [$Cell.H2O2] |  |
| **3** |  | time [$Cell.OHr]         K1 [$Cell.H2O2] [$Cell.c\_LIP]     K1 [$Cell.H2O2] [$Cell.m\_Fe2]     K4 [$Cell.OHr] [$Cell.H2O2] |  |
| **4** |  | time [$Cell.O\_R]       K2 [$Cell.O\_R] [$Cell.Fe3]     K4 [$Cell.OHr] [$Cell.H2O2] |  |
| **5** |  | time [$Cell.c\_LIP]       K1 [$Cell.H2O2] [$Cell.c\_LIP]     K2 [$Cell.O\_R] [$Cell.Fe3] |  |
| **6** |  | time [$Cell.m\_Fe2]     K1 [$Cell.H2O2] [$Cell.m\_Fe2] |  |

  
  
