# Supplementary material for: A Systems Biology Approach Towards a Comprehensive Understanding of Ferroptosis
Source: Int J Mol Sci. 2024 Nov 2;25(21):11782. doi: 10.3390/ijms252111782 (PMC11546516; doi:10.3390/ijms252111782)
Supplement: Supplementary file 1 [file ijms-25-11782-s001.zip › Kinetic equations/GSH synthesis.html]

Differential equation system  
  

|  |  |  |  |
| --- | --- | --- | --- |
| **1** |  | time [$Cell.Cysteine]             Kc1 [$Cell.GCL] [$Cell.Cysteine] [$Cell.Glutamate\_1]     1.0   [$Cell.Cysteine] Kcyst   [$Cell.Glutamate\_1] Kglut       KcTXNRD [$Cell.TXNRD1] [$Cell.Cystine\_1]   Kmcyst [$Cell.Cystine\_1]   KtCys [$extracellular\_space.Cysteine\_ex] |  |
| **2** |  | time [$Cell.Cystine\_1]         KcTXNRD [$Cell.TXNRD1] [$Cell.Cystine\_1]   Kmcyst [$Cell.Cystine\_1]         KcXC [$cell\_membrane.System\_Xc] [$Cell.Glutamate\_1] [$extracellular\_space.Cystine]     KmXCglu [$Cell.Glutamate\_1]   KmXCcys [$extracellular\_space.Cystine] |  |
| **3** |  | time [$Cell.GGC]           Kc1 [$Cell.GCL] [$Cell.Cysteine] [$Cell.Glutamate\_1]     1.0   [$Cell.Cysteine] Kcyst   [$Cell.Glutamate\_1] Kglut         KcGSS [$Cell.GSH\_synthetase] [$Cell.GGC] [$Cell.Glycine\_1]     1.0   [$Cell.Glycine\_1] Kglyc   [$Cell.GGC] Kggc |  |
| **4** |  | time [$Cell.GSH]           KcGSS [$Cell.GSH\_synthetase] [$Cell.GGC] [$Cell.Glycine\_1]     1.0   [$Cell.Glycine\_1] Kglyc   [$Cell.GGC] Kggc   KtGSH [$Cell.GSH] |  |
| **5** |  | time [$Cell.Glutamate\_1]           Kc1 [$Cell.GCL] [$Cell.Cysteine] [$Cell.Glutamate\_1]     1.0   [$Cell.Cysteine] Kcyst   [$Cell.Glutamate\_1] Kglut         KcXC [$cell\_membrane.System\_Xc] [$Cell.Glutamate\_1] [$extracellular\_space.Cystine]     KmXCglu [$Cell.Glutamate\_1]   KmXCcys [$extracellular\_space.Cystine] |  |
| **6** |  | time [$Cell.Glycine\_1]           KcGSS [$Cell.GSH\_synthetase] [$Cell.GGC] [$Cell.Glycine\_1]     1.0   [$Cell.Glycine\_1] Kglyc   [$Cell.GGC] Kggc   KtGly [$extracellular\_space.Glycine] |  |
| **1** |  | time [$extracellular\_space.AA]           V4 [$extracellular\_space.Cystine] [$extracellular\_space.GSH\_1] [$extracellular\_space.AA]       K4 [$extracellular\_space.Cystine] [$extracellular\_space.GSH\_1] [$extracellular\_space.AA]       Kc1 [$extracellular\_space.AA] [$extracellular\_space.Glutamate]     1.0   [$extracellular\_space.AA] Kcyst   [$extracellular\_space.Glutamate] Kglut |  |
| **2** |  | time [$extracellular\_space.Cysteine\_ex]     KtCys [$extracellular\_space.Cysteine\_ex]       KcGGT [$extracellular\_space.GGT] [$extracellular\_space.Cysteinylglycine]   K53 [$extracellular\_space.Cysteinylglycine] |  |
| **3** |  | time [$extracellular\_space.Cysteinylglycine]           V4 [$extracellular\_space.Cystine] [$extracellular\_space.GSH\_1] [$extracellular\_space.AA]       K4 [$extracellular\_space.Cystine] [$extracellular\_space.GSH\_1] [$extracellular\_space.AA]       KcGGT [$extracellular\_space.GGT] [$extracellular\_space.Cysteinylglycine]   K53 [$extracellular\_space.Cysteinylglycine] |  |
| **4** |  | time [$extracellular\_space.Cystine]           V4 [$extracellular\_space.Cystine] [$extracellular\_space.GSH\_1] [$extracellular\_space.AA]       K4 [$extracellular\_space.Cystine] [$extracellular\_space.GSH\_1] [$extracellular\_space.AA]         KcXC [$cell\_membrane.System\_Xc] [$Cell.Glutamate\_1] [$extracellular\_space.Cystine]     KmXCglu [$Cell.Glutamate\_1]   KmXCcys [$extracellular\_space.Cystine] |  |
| **5** |  | time [$extracellular\_space.GSH\_1]     KtGSH [$Cell.GSH]         V4 [$extracellular\_space.Cystine] [$extracellular\_space.GSH\_1] [$extracellular\_space.AA]       K4 [$extracellular\_space.Cystine] [$extracellular\_space.GSH\_1] [$extracellular\_space.AA] |  |
| **6** |  | time [$extracellular\_space.Glutamate]           Kc1 [$extracellular\_space.AA] [$extracellular\_space.Glutamate]     1.0   [$extracellular\_space.AA] Kcyst   [$extracellular\_space.Glutamate] Kglut   K8 [$extracellular\_space.\_5\_oxoproline]         KcXC [$cell\_membrane.System\_Xc] [$Cell.Glutamate\_1] [$extracellular\_space.Cystine]     KmXCglu [$Cell.Glutamate\_1]   KmXCcys [$extracellular\_space.Cystine] |  |
| **7** |  | time [$extracellular\_space.Glycine]     KtGly [$extracellular\_space.Glycine]       KcGGT [$extracellular\_space.GGT] [$extracellular\_space.Cysteinylglycine]   K53 [$extracellular\_space.Cysteinylglycine] |  |
| **8** |  | time [$extracellular\_space.\_5\_oxoproline]     K8 [$extracellular\_space.\_5\_oxoproline]     VmGGCT [$extracellular\_space.y\_glu\_AA]   KmGGCT [$extracellular\_space.y\_glu\_AA] |  |
| **9** |  | time [$extracellular\_space.y\_glu\_AA]             V4 [$extracellular\_space.Cystine] [$extracellular\_space.GSH\_1] [$extracellular\_space.AA]       K4 [$extracellular\_space.Cystine] [$extracellular\_space.GSH\_1] [$extracellular\_space.AA]       Kc1 [$extracellular\_space.AA] [$extracellular\_space.Glutamate]     1.0   [$extracellular\_space.AA] Kcyst   [$extracellular\_space.Glutamate] Kglut     VmGGCT [$extracellular\_space.y\_glu\_AA]   KmGGCT [$extracellular\_space.y\_glu\_AA] |  |

  
  
