# Supplementary material for: A Systems Biology Approach Towards a Comprehensive Understanding of Ferroptosis
Source: Int J Mol Sci. 2024 Nov 2;25(21):11782. doi: 10.3390/ijms252111782 (PMC11546516; doi:10.3390/ijms252111782)
Supplement: Supplementary file 1 [file ijms-25-11782-s001.zip › Kinetic equations/Iron Metabolism.html]

Differential equation system  
  

|  |  |  |  |
| --- | --- | --- | --- |
| **1** |  | time [$Cell.Fe3\_3]       KdFT [$Cell.Fe3\_3] [$Cell.Ferritin]     KaFT [$Cell.Ferritin] [$Cell.Fe3\_3] |  |
| **2** |  | time [$Cell.Ferritin]       KdFT [$Cell.Fe3\_3] [$Cell.Ferritin]     KaFT [$Cell.Ferritin] [$Cell.Fe3\_3] |  |
| **3** |  | time [$Cell.Ferritin\_Fe]       KdFT [$Cell.Fe3\_3] [$Cell.Ferritin]     KaFT [$Cell.Ferritin] [$Cell.Fe3\_3] |  |
| **4** |  | time [$Cell.cytosolic\_Labile\_Iron\_Pool]             K16 [$Cell.cytosolic\_Labile\_Iron\_Pool] [$Cell.mitochondrion.MCU]     V17 [$Cell.lysosome.Fe2\_1]   K17 [$Cell.lysosome.Fe2\_1]     K15 [$Cell.lysosome.ZIP8\_14] [$extracellular\_space.Fe2]     K15 [$Cell.lysosome.ZIP8\_14] [$extracellular\_space.Fe2]     V19 [$Cell.lysosome.Fe2\_1]   K19 [$Cell.lysosome.Fe2\_1] |  |
| **1** |  | time [$Cell.lysosome.Fe2\_1]         V17 [$Cell.lysosome.Fe2\_1]   K17 [$Cell.lysosome.Fe2\_1]     K15 [$Cell.lysosome.ZIP8\_14] [$extracellular\_space.Fe2]     V1 [$Cell.lysosome.Fe3\_1]   K1 [$Cell.lysosome.Fe3\_1] |  |
| **2** |  | time [$Cell.lysosome.Fe3\_1]       K13 [$cell\_membrane.TF\_TFR1]   [$Cell.lysosome.Fe3\_1] 2.0     V1 [$Cell.lysosome.Fe3\_1]   K1 [$Cell.lysosome.Fe3\_1] |  |
| **1** |  | time [$Cell.mitochondrion.Mitochondrial\_Ferritin]       KaFT [$Cell.mitochondrion.Mitochondrial\_Ferritin] [$Cell.mitochondrion.mitochondrial\_\_Labile\_Iron\_Pool]     KdFT [$Cell.mitochondrion.Mitochondrial\_Ferritin] [$Cell.mitochondrion.mitochondrial\_\_Labile\_Iron\_Pool] |  |
| **2** |  | time [$Cell.mitochondrion.Mitochondrial\_Ferritin\_Fe]       KaFT [$Cell.mitochondrion.Mitochondrial\_Ferritin] [$Cell.mitochondrion.mitochondrial\_\_Labile\_Iron\_Pool]     KdFT [$Cell.mitochondrion.Mitochondrial\_Ferritin] [$Cell.mitochondrion.mitochondrial\_\_Labile\_Iron\_Pool] |  |
| **3** |  | time [$Cell.mitochondrion.mitochondrial\_\_Labile\_Iron\_Pool]         K16 [$Cell.cytosolic\_Labile\_Iron\_Pool] [$Cell.mitochondrion.MCU]     KaFT [$Cell.mitochondrion.Mitochondrial\_Ferritin] [$Cell.mitochondrion.mitochondrial\_\_Labile\_Iron\_Pool]     KdFT [$Cell.mitochondrion.Mitochondrial\_Ferritin] [$Cell.mitochondrion.mitochondrial\_\_Labile\_Iron\_Pool] |  |
| **1** |  | time [$cell\_membrane.TF\_TFR1]       K12 [$cell\_membrane.Transferrin\_Receptor] [$extracellular\_space.Transferrin\_1]     K13 [$cell\_membrane.TF\_TFR1]   [$Cell.lysosome.Fe3\_1] 2.0 |  |
| **2** |  | time [$cell\_membrane.TF\_TFR1\_1]       K13 [$cell\_membrane.TF\_TFR1]   [$Cell.lysosome.Fe3\_1] 2.0   K14 [$cell\_membrane.TF\_TFR1] |  |
| **3** |  | time [$cell\_membrane.Transferrin\_Receptor]       K12 [$cell\_membrane.Transferrin\_Receptor] [$extracellular\_space.Transferrin\_1]   K14 [$cell\_membrane.TF\_TFR1] |  |
| **1** |  | time [$extracellular\_space.Fe2]         K15 [$Cell.lysosome.ZIP8\_14] [$extracellular\_space.Fe2]     V19 [$Cell.lysosome.Fe2\_1]   K19 [$Cell.lysosome.Fe2\_1]   V9 [$extracellular\_space.Fe2] |  |
| **2** |  | time [$extracellular\_space.Fe3]       K11 [$extracellular\_space.Transferrin]   [$extracellular\_space.Fe3] 2.0   V9 [$extracellular\_space.Fe2] |  |
| **3** |  | time [$extracellular\_space.Transferrin]     K14 [$cell\_membrane.TF\_TFR1]     K11 [$extracellular\_space.Transferrin]   [$extracellular\_space.Fe3] 2.0 |  |
| **4** |  | time [$extracellular\_space.Transferrin\_1]       K12 [$cell\_membrane.Transferrin\_Receptor] [$extracellular\_space.Transferrin\_1]     K11 [$extracellular\_space.Transferrin]   [$extracellular\_space.Fe3] 2.0 |  |

  
  
