# Supplementary material for: A Systems Biology Approach Towards a Comprehensive Understanding of Ferroptosis
Source: Int J Mol Sci. 2024 Nov 2;25(21):11782. doi: 10.3390/ijms252111782 (PMC11546516; doi:10.3390/ijms252111782)
Supplement: Supplementary file 1 [file ijms-25-11782-s001.zip › Kinetic equations/Lipid Peroxidation.html]

Differential equation system  
  

|  |  |  |  |
| --- | --- | --- | --- |
| **1** |  | time [$Cell.Fe3]         k10 [$Cell.PUFAs\_O\_OH] [$Cell.Fe3]     K4 [$Cell.c\_LIP] [$Cell.PUFAs\_O\_OH]   k8 [$Cell.PUFAs\_O\_OH] |  |
| **2** |  | time [$Cell.HOr]     K1 [$Cell.HOr] [$Cell.PE\_PUFAs] |  |
| **3** |  | time [$Cell.LOOR]         K3 [$Cell.LR]     k10 [$Cell.PUFAs\_O\_OH] [$Cell.Fe3]     K7 [$Cell.PE\_PUFAs] [$Cell.LOOR]   K9   [$Cell.LOOR] 2.0 |  |
| **4** |  | time [$Cell.LR]             K2 [$Cell.PE\_PUFAs] [$Cell.PUFAs\_OR]     K1 [$Cell.HOr] [$Cell.PE\_PUFAs]   K3 [$Cell.LR]     K6 [$Cell.PE\_PUFAs] [$Cell.OLOOR]     K7 [$Cell.PE\_PUFAs] [$Cell.LOOR] |  |
| **5** |  | time [$Cell.OLOOH]     K6 [$Cell.PE\_PUFAs] [$Cell.OLOOR] |  |
| **6** |  | time OLOOR       K4 [$Cell.c\_LIP] [$Cell.PUFAs\_O\_OH]     K6 [$Cell.PE\_PUFAs] [$Cell.OLOOR] |  |
| **7** |  | time [$Cell.PE\_PUFAs]             K2 [$Cell.PE\_PUFAs] [$Cell.PUFAs\_OR]     K1 [$Cell.HOr] [$Cell.PE\_PUFAs]     K5 [$Cell.PE\_PUFAs] [$Cell.LOXs]     K6 [$Cell.PE\_PUFAs] [$Cell.OLOOR]     K7 [$Cell.PE\_PUFAs] [$Cell.LOOR] |  |
| **8** |  | time [$Cell.PE\_PUFAs\_OH]       K2 [$Cell.PE\_PUFAs] [$Cell.PUFAs\_OR]   K9   [$Cell.LOOR] 2.0 |  |
| **9** |  | time [$Cell.PUFAs\_OR]     k8 [$Cell.PUFAs\_O\_OH]     K2 [$Cell.PE\_PUFAs] [$Cell.PUFAs\_OR] |  |
| **10** |  | time [$Cell.PUFAs\_O\_OH]             k10 [$Cell.PUFAs\_O\_OH] [$Cell.Fe3]     K4 [$Cell.c\_LIP] [$Cell.PUFAs\_O\_OH]     K5 [$Cell.PE\_PUFAs] [$Cell.LOXs]     K7 [$Cell.PE\_PUFAs] [$Cell.LOOR]   k8 [$Cell.PUFAs\_O\_OH] |  |
| **11** |  | time [$Cell.c\_LIP]         k10 [$Cell.PUFAs\_O\_OH] [$Cell.Fe3]     K4 [$Cell.c\_LIP] [$Cell.PUFAs\_O\_OH]   k8 [$Cell.PUFAs\_O\_OH] |  |

  
  
