# Supplementary material for: A Systems Biology Approach Towards a Comprehensive Understanding of Ferroptosis
Source: Int J Mol Sci. 2024 Nov 2;25(21):11782. doi: 10.3390/ijms252111782 (PMC11546516; doi:10.3390/ijms252111782)
Supplement: Supplementary file 1 [file ijms-25-11782-s001.zip › Kinetic equations/Lipid Synthesis.html]

Differential equation system  
  

|  |  |  |  |
| --- | --- | --- | --- |
| **1** |  | time $Cell.CoA     V1     $Cell.PUFAs $Cell.CoA K1   1.0     $Cell.PUFAs $Cell.CoA K1 |  |
| **2** |  | time $Cell.CoA\_PUFAs       V1     $Cell.PUFAs $Cell.CoA K1   1.0     $Cell.PUFAs $Cell.CoA K1     V2     $Cell.CoA\_PUFAs $Cell.LysoPE K2   1.0     $Cell.CoA\_PUFAs $Cell.LysoPE K2 |  |
| **3** |  | time $Cell.LysoPE     V2     $Cell.CoA\_PUFAs $Cell.LysoPE K2   1.0     $Cell.CoA\_PUFAs $Cell.LysoPE K2 |  |
| **4** |  | time $Cell.PE\_PUFAs     V2     $Cell.CoA\_PUFAs $Cell.LysoPE K2   1.0     $Cell.CoA\_PUFAs $Cell.LysoPE K2 |  |
| **5** |  | time $Cell.PUFAs     V1     $Cell.PUFAs $Cell.CoA K1   1.0     $Cell.PUFAs $Cell.CoA K1 |  |

  
  
