# Supplementary material for: A Systems Biology Approach Towards a Comprehensive Understanding of Ferroptosis
Source: Int J Mol Sci. 2024 Nov 2;25(21):11782. doi: 10.3390/ijms252111782 (PMC11546516; doi:10.3390/ijms252111782)
Supplement: Supplementary file 1 [file ijms-25-11782-s001.zip › Kinetic equations/Pentose Phosphate Pathway.html]

Differential equation system  
  

|  |  |  |  |
| --- | --- | --- | --- |
| **1** |  | time [$Cell.Glucose\_6\_phosphate]     V1   [$Cell.Glucose\_6\_phosphate] K1   1.0   [$Cell.Glucose\_6\_phosphate] K1 |  |
| **2** |  | time [$Cell.NADPH]       V2     [$Cell.\_6\_Phosphogluconate] [$Cell.NADP\_p] K2   1.0     [$Cell.\_6\_Phosphogluconate] [$Cell.NADP\_p] K2     V1   [$Cell.Glucose\_6\_phosphate] K1   1.0   [$Cell.Glucose\_6\_phosphate] K1 |  |
| **3** |  | time [$Cell.NADP\_p]       V2     [$Cell.\_6\_Phosphogluconate] [$Cell.NADP\_p] K2   1.0     [$Cell.\_6\_Phosphogluconate] [$Cell.NADP\_p] K2     V1   [$Cell.Glucose\_6\_phosphate] K1   1.0   [$Cell.Glucose\_6\_phosphate] K1 |  |
| **4** |  | time [$Cell.Rebulose\_5\_phosphate]     V2     [$Cell.\_6\_Phosphogluconate] [$Cell.NADP\_p] K2   1.0     [$Cell.\_6\_Phosphogluconate] [$Cell.NADP\_p] K2 |  |
| **5** |  | time [$Cell.\_6\_Phosphogluconate]       V2     [$Cell.\_6\_Phosphogluconate] [$Cell.NADP\_p] K2   1.0     [$Cell.\_6\_Phosphogluconate] [$Cell.NADP\_p] K2     V1   [$Cell.Glucose\_6\_phosphate] K1   1.0   [$Cell.Glucose\_6\_phosphate] K1 |  |

  
  
