# Supplementary material for: A Systems Biology Approach Towards a Comprehensive Understanding of Ferroptosis
Source: Int J Mol Sci. 2024 Nov 2;25(21):11782. doi: 10.3390/ijms252111782 (PMC11546516; doi:10.3390/ijms252111782)
Supplement: Supplementary file 1 [file ijms-25-11782-s001.zip › Supplementary materials.pdf]

“Fenton reaction” module

Table S1. Model parameter values for reactions in the Module “Fenton reaction”

Reaction	Value	Ref
$Fe^{2+} + H_2O_2 \rightarrow Fe^{3+} + \cdot OH + OH-$	$5.7 * 10^2$ $M^{-1}s^{-1}$	[1]
$Fe^{3+} + \cdot O_2^- \rightarrow Fe^{2+} + O_2$<br>$Fe^{2+} + H_2O_2 \rightarrow Fe^{3+} + OH^- + \bullet OH$	$3.1 * 10^5$ $M^{-1}s^{-1}$	[1]

1. Kaźmierczak-Barańska J, Boguszewska K, Adamus-Grabicka A, Karwowski BT. **Two Faces of Vitamin C-Antioxidative and Pro-Oxidative Agent**. Nutrients. 2020 May 21;12(5):1501. doi: 10.3390/nu12051501. PMID: 32455696; PMCID: PMC7285147.

“Iron Metabolism” module

Table S2. Model parameter values for reactions in the Module “Iron Metabolism”

Compartment/Reaction	Value	Ref
**Extracellular space**		
Fe^{3+} -Ceruloplasmin → Fe^{2+}	Rate of oxidation of the Cu^{1+} to Cu^{2+} in ceruloplasmin by O2 μM^{-1} min^{-1} 5.93E+0<br>Rate coefficient of oxidation of Fe^{2+} to Fe^{3+} by ceruloplasmin μM^{-1} min^{-1} 8.99E+1 (1.5 s)	[1]
Fe^{3+} + Transferrin → Transferrin__Fe	K C-domain = 4,7 × 10^{20} M^{-1} and K N-domain = 2,4 × 10^{19} M^{-1} for C-domain and N-domain respectively<br>$K_d = 10^{-23}$ M	[2,3,4,14]
**Cell Membrane**		
Transferrin_1 + Transferrin_Receptor → TF_TFR1	Rate constant Association rate of HT and R proteins 1.7×10^4 M^{-1} s^{-1}	[5,12]
TF_TFR1 → Fe^{3+}_1 + TF_TFR1_1	Rate coefficient of dissociation of (Fe^{3+}) Tf from complex with TfR µM 3.38E-2<br>1.125 × 10^{-3} s^{-1}	[6, 16]
TF_TFR1_1 → Transferrin_Receptor + Transferrin	Kd = 5 × 10^{-9} M Ph 7.4 K562 cells	[4]
Ferroportin_ Fe^{3+} → Fe^{3+} + Ferroportin	K_m of 1.9 ± 0.26 µM and V_{max} of 0.13 ± 0.09 min^{-1}<br>4.3 × 10^{-2} s^{-1}	[13, 16]
Fe^{2+} -ZIP8/14 → Fe^{2+}	VmaxFe = 1,0 ± 0,1 pmol/min, KdCa = 0,39 ± 0,12 mM<br>0,00000000015 mM/s	[7,15]
**Cell**		
Fe + Ferritin → Ferritin_Fe	$K_{d1} = 0.2$; $K_{d2} = 5.3$	[8]
**Cell (Lysosome)**		
Fe^{3+} -STEAP3/4 → Fe^{2+}	$V_{max} = 3.0$ (0.05 s); $K_m = 5.0$	[9]
Fe^{2+} -DMT1 → Fe^{2+}	$V_{max} = 2.2193$ (0,0369883 s); $K_m = 1.22$	[10,16]
Fe^{2+} -ZIP8/14 → Fe^{2+}	VmaxFe = 1,0 ± 0,1 pmol/min, KdCa = 0,39 ± 0,12 mM<br>0,00000000015 mM/s	[7,15]
**Cell (Mitochondrion)**		
Fe^{2+} -MCU → Fe^{2+}	Dissociation constant for Ca^{2+} translocation by the MCU 6 µM	[11]

	Dissociation constant for MCU activation by Ca^{2+} 0.38 μM<br>Rate constant of the MCU 0.0006 μM.s−1	

1. Sarkar J, Potdar AA, Saidel GM. **Whole-body iron transport and metabolism: Mechanistic, multi-scale model to improve treatment of anemia in chronic kidney disease**. PLoS Comput Biol. 2018 Apr 16;14(4):e1006060. doi: 10.1371/journal.pcbi.1006060. PMID: 29659573; PMCID: PMC5919696.

2. Thorstensen and I. Romslo. **The role of transferrin in the mechanism of cellular iron uptake**. The Biochemical Journal, 271(1):1–9, October 1990. ISSN 0264-6021. URL http://view.ncbi.nlm.nih.gov/pubmed/2222403].

3. Kaplan. **Mechanisms of cellular iron acquisition: another iron in the fire**. Cell, 111 (5):603–606, November 2002. ISSN 0092-8674. URL http://view.ncbi.nlm.nih.gov/pubmed/12464171

4. D. R. Richardson and P. Ponka. **The molecular mechanisms of the metabolism and transport of iron in normal and neoplastic cells**. Biochimica et Biophysica Acta, 1331(1): 1–40, March 1997. ISSN 0006-3002. URL http://view.ncbi.nlm.nih.gov/pubmed/9325434

5. J. Lebron. **Crystal Structure of the Hemochromatosis Protein HFE and Characterization of Its Interaction with Transferrin Receptor**. Cell, 93(1):111–123, April 1998. ISSN 00928674. doi: 10.1016/S0092-8674(00)81151-4. URL http://dx.doi.org/10.1016/S0092-8674(00)81151-4.

6. Pootrakul P, Christensen A, Josephson B, Finch CA. **Role of transferrin in determining internal iron distribution**. Blood. 1977; 49(6):957–66. PMID: 861377.

7. van Raaij SEG, Srai SKS, Swinkels DW, van Swelm RPL. **Iron uptake by ZIP8 and ZIP14 in human proximal tubular epithelial cells**. Biometals. 2019 Apr;32(2):211-226. doi: 10.1007/s10534-019-00183-7. Epub 2019 Feb 26. PMID: 30806852; PMCID: PMC6437295.

8. Leidgens S, Bullough KZ, Shi H, Li F, Shakoury-Elizeh M, Yabe T, Subramanian P, Hsu E, Natarajan N, Nandal A, Stemmler TL, Philpott CC. **Each member of the poly-r(C)-binding protein 1 (PCBP) family exhibits iron chaperone activity toward ferritin**. J Biol Chem. 2013 Jun 14;288(24):17791-802. doi: 10.1074/jbc.M113.460253. Epub 2013 May 2. PMID: 23640898; PMCID: PMC3682578.

9. Kleven MD, Dlakić M, Lawrence CM. **Characterization of a single b-type heme, FAD, and metal binding sites in the transmembrane domain of six-transmembrane epithelial antigen of the prostate (STEAP) family proteins**. J Biol Chem. 2015 Sep 11;290(37):22558-69. doi: 10.1074/jbc.M115.664565. Epub 2015 Jul 23. PMID: 26205815; PMCID: PMC4566230.

10. Cegarra L, Colins A, Gerdtzen ZP, Nuñez MT, Salgado JC. **Mathematical modeling of the relocation of the divalent metal transporter DMT1 in the intestinal iron absorption process**. PLoS One. 2019 Jun 10;14(6):e0218123. doi: 10.1371/journal.pone.0218123. PMID: 31181103; PMCID: PMC6557526.

11. Wacquier, B., Combettes, L., Van Nhieu, G. et al. **Interplay Between Intracellular Ca2+ Oscillations and Ca2+-stimulated Mitochondrial Metabolism**. Sci Rep 6, 19316 (2016). https://doi.org/10.1038/srep19316

12. Khan AI, Liu J, Dutta P. **Iron transport kinetics through blood-brain barrier endothelial cells**. Biochim Biophys Acta Gen Subj. 2018 May;1862(5):1168-1179. doi: 10.1016/j.bbagen.2018.02.010. Epub 2018 Feb 18. PMID: 29466707; PMCID: PMC5866250.

13. Pan, Y., Ren, Z., Gao, S. et al. **Structural basis of ion transport and inhibition in ferroportin**. Nat Commun 11, 5686 (2020). https://doi.org/10.1038/s41467-020-19458-6

14. Quarles, C.D., Marcus, R.K. & Brumaghim, J.L. **Competitive binding of Fe3+, Cr3+, and Ni2+ to transferrin**. J Biol Inorg Chem 16, 913–921 (2011). https://doi.org/10.1007/s00775-011-0792-9

15. Pinilla-Tenas J. J. et al. **Zip14 is a complex broad-scope metal-ion transporter whose functional properties support roles in the cellular uptake of zinc and nontransferrin-bound iron** //American Journal of Physiology-Cell Physiology. – 2011. – T. 301. – №. 4. – C. C862-C871.

16. Khan AI, Liu J, Dutta P. **Iron transport kinetics through blood-brain barrier endothelial cells**. Biochim Biophys Acta Gen Subj. 2018 May;1862(5):1168-1179. doi: 10.1016/j.bbagen.2018.02.010. Epub 2018 Feb 18. PMID: 29466707; PMCID: PMC5866250.

“Lipid Synthesis” module

Table S3. Model parameter values for reactions in the Module “Lipid Synthesis”

Reaction	Value	Ref
CoA + PUFAs (Cat.ACSL4) → CoA-PUFAs	Km = 6.7 Vmax = 3444	[1,3]
CoA-PUFAs + LysoPE (Cat.LPCAT3) → PE-PUFAs	Km = 71.56 Vmax = 6,247	[2,3]

1. Stinnett L, Lewin TM, Coleman RA. **Mutagenesis of rat acyl-CoA synthetase 4 indicates amino acids that contribute to fatty acid binding**. Biochim Biophys Acta. 2007 Jan;1771(1):119-25. doi: 10.1016/j.bbalip.2006.09.016. Epub 2006 Oct 6. PMID: 17110164; PMCID: PMC1828365.

2. Zhao Y, Chen YQ, Bonacci TM, Bredt DS, Li S, Bensch WR, Moller DE, Kowala M, Konrad RJ, Cao G. **Identification and characterization of a major liver lysophosphatidylcholine acyltransferase**. J Biol Chem. 2008 Mar 28;283(13):8258-65. doi: 10.1074/jbc.M710422200. Epub 2008 Jan 14. PMID: 18195019.

3. Akberdin, I.R.; Kiselev, I.N.; Pintus, S.S.; Sharipov, R.N.; Vertyshev, A.Y.; Vinogradova, O.L.; Popov, D.V.; Kolpakov, F.A. **A Modular Mathematical Model of Exercise-Induced Changes in Metabolism, Signaling, and Gene Expression in Human Skeletal Muscle**. Int. J. Mol. Sci. 2021, 22, 10353. https://doi.org/10.3390/ijms221910353

“Lipid peroxidation” module

Table S4. Model parameter values for reactions in the Module “Lipid peroxidation”

Reaction	Value	Ref
HO* + PE-PUFAs → PE-PUFAs*	100,000,000,000.0000000	[1]
PE-PUFAs + PE-PUFAs-O* → PE-PUFAs* + PE-PUFAs-OH	12,600,000.0000000	[1]
PE-PUFAs* → PE-PUFAs-OO*	1,000,000,000.0000000	[2]
PE-PUFAs-OOH + Fe2+ → Fe3+ + O-PE-PUFAs-OO*	3,500,000.0000000	[3]
PE-PUFAs (Cat.LOXs) → PE-PUFAs-OOH	0.0600000	[4]
O-PE-PUFAs-OO* + PE-PUFAs → PE-PUFAs* + O-PE-PUFAs-OOH	70.0000000	[2]

PE-PUFAs-OO* + PE-PUFAs → PE-PUFAs* + PE-PUFAs-OOH	70.0000000	[2]
PE-PUFAs-OOH + Fe2+ → PE-PUFAs-O* + Fe3+	320.0000000	[5]
2PE-PUFAs-OO* → PE-PUFAs-OH	100,000.0000000	[2]

1. Tavadyan, L., Khachoyan, A., Martoyan, G., & Kamal-Eldin, A. (2007). **Numerical revelation of the kinetic significance of individual steps in the reaction mechanism of methyl linoleate peroxidation inhibited by α-tocopherol**. Chemistry and Physics of Lipids, 147(1), 30-45.

2. Pliss E. M. et al. **Kinetic model of polyunsaturated fatty acids oxidation in micelles** //Chemistry and Physics of Lipids. – 2021. – T. 237. – C. 105089.

3. Pratt DA, Tallman KA, Porter NA. **Free radical oxidation of polyunsaturated lipids: New mechanistic insights and the development of peroxyl radical clocks**. Acc Chem Res. 2011 Jun 21;44(6):458-67. doi: 10.1021/ar200024c. Epub 2011 Apr 12. PMID: 21486044; PMCID: PMC3124811.

4. Schöneich C. et al. **Oxidation of polyunsaturated fatty acids and lipids through thiyl and sulfonyl radicals: reaction kinetics, and influence of oxygen and structure of thiyl radicals** //Archives of biochemistry and biophysics. – 1992. – T. 292. – №. 2. – C. 456-467.

5. Antunes F. et al. **Lipid peroxidation in mitochondrial inner membranes**. **I. An integrative kinetic model** //Free Radical Biology and Medicine. – 1996. – T. 21. – №. 7. – C. 917-943.

“Pentose phosphate pathway” module

Table S5. Model parameter values for reactions in the Module “Pentose phosphate pathway”

Reaction	Value	Ref
**6-phosphogluconate dehydrogenase (GND1, GND2)**<br>$NADP^+$ + 6-Phosphogluconate (Cat. PGD) → NADPH + Rebulose-5-phosphate	Kcat 28.0 s^{-1}<br>Kp6g 0.062 mM<br>Knadp 0.094 mM<br>Kru5p 0.1 mM<br>Knadph 0.055 mM	[1]
**Glucose-6-phosphate dehydrogenase (ZWF1)**<br>Glucose-6-phosphate (Cat. G6PD) → NADPH + $NADP^+$ + 6-Phosphogluconate	Kcat 189 s^{-1}<br>Kg6p 0.042 mM<br>Knadp 0.045 mM<br>Kg6l 0.1 mM<br>Knadph 0.017 mM	[1]

1. Messiha HL, Kent E, Malys N, Carroll KM, Swainston N, Mendes P, Smallbone K. 2014. **Enzyme characterisation and kinetic modelling of the pentose phosphate pathway in yeast**. PeerJ PrePrints 2:e146v4 https://doi.org/10.7287/peerj.preprints.146v4

“Antioxidant system” module

Table S6. Model parameter values for reactions in the Module “Antioxidant system”

Reaction	Value	Ref
GSH + PE-PUFAs-O-OH (Cat.GPx4) → GSSH + PE-PUFAs-OH	21 000 000,0000000	[1]
GSSH + NADPH (Cat.GR) → GSH + NADP+	0,0000500	[2]
GSH + PE-PUFAs-O* (Cat.GPx4) → PE-PUFAs-O-OH + GSSH	21 000 000,0000000	[1]
GSH + H2O2 (Cat.GPx4) → GSSH + H2O	180 000 000,0000000	[4]
GSH + O* (Cat.GPx4) → GSSH + O2	150 000,0000000	[5]
O-PE-PUFAs-OO* + aT-OH → O-PE-PUFAs-OOH + aT-O*	1 850 000,0000000	[6]
aT-O* (Cat.Ascorbate) → aT-OH	200 000,0000000	[7]

1. Antunes F, Salvador A, Pinto RE. **PHGPx and phospholipase A2/GPx: comparative importance on the reduction of hydroperoxides in rat liver mitochondria**. Free Radic Biol Med. 1995 Nov;19(5):669-77. doi: 10.1016/0891-5849(95)00040-5. PMID: 8529927.

2. Antunes F. et al. **Lipid peroxidation in mitochondrial inner membranes. I. An integrative kinetic model** //Free Radical Biology and Medicine. – 1996. – T. 21. – №. 7. – C. 917-943.

4. Flohé L, Loschen G, Günzler WA, Eichele E. **Glutathione peroxidase, V. The kinetic mechanism**. Hoppe Seylers Z Physiol Chem. 1972 Jun;353(6):987-99. doi: 10.1515/bchm2.1972.353.1.987. PMID: 5066111.

5. Scarpa M. et al. **Activated oxygen species in the oxidation of glutathione A kinetic study** //Biophysical Chemistry. – 1996. – T. 60. – №. 1-2. – C. 53-61.

6. Tavadyan L, Khachoyan A, Martoyan G, Kamal-Eldin A. **Numerical revelation of the kinetic significance of individual steps in the reaction mechanism of methyl linoleate peroxidation inhibited by alpha-tocopherol**. Chem Phys Lipids. 2007 May;147(1):30-45. doi: 10.1016/j.chemphyslip.2007.03.002. Epub 2007 Mar 12. PMID: 17445788.

7. Buettner GR. **The pecking order of free radicals and antioxidants: lipid peroxidation, alpha-tocopherol, and ascorbate**. Arch Biochem Biophys. 1993 Feb 1;300(2):535-43. doi: 10.1006/abbi.1993.1074. PMID: 8434935.

“GSH Synthesis” module

Table S7. Model parameter values for reactions in the Module “GSH Synthesis module”

Reaction	Value	Ref
Cysteinylglycine (Cat. Peptidase) → Cycteine + Glycine	(Mn^{2+}) K_m = 0.4 mM, K_{cat} = 86.5 s^{-1}; (Zn^{2+}) K_m = 0.8 mM, K_{cat} = 76.9 s^{-1}	[4]
Cyctine + GSH + AA (Cat.GGT) → Cysteinylglycine + γ-glu-AA	V_{max} = 1200 min (20 s)<br>g-Glutamyl (K_m = 1.4 mM)<br>Glycylglycine (K_m = 10 mM)	[1,2]
Glutamate + AA (Cat.GCL) → γ-glu-AA	K_{cat} = 6.1 min^{-1} (0.1 s); K_m = 9.1 mM	[6]
5-oxoproline → Glutamate	K_m = 51 mM; V_{max} = 167 mM/h (0.046 s)	[5]
γ-glu-AA (Cat.GGCT) → 5-oxoproline	K_m = 3.13 mM; V_{max} = 980 mmol/(h*mg) (0.272 s)	[3]
Cysteine + Glutamate -GSH, GCL→ GGC	0.1016 s	[7]
GGC + Glycine -GSS → GSH	V_{max} = 7.91 s	[8]
Cystine -TXNRD1→ Cysteine	K_m = 0.8, k_{cat} = 1.8 min^{-1} (0.03 s)	[9,10]
GSH → GSH	V(cytosole-bloode) = 1152 h (0.32 s), V(blood-outside) = 9 h (0.00247 s)	[11]
Cystine + Glutamate -System_Xc-→ Cystine + Glutamate	K_i for glutamate inhibition of cystine uptake =150 mM<br>K_i for cystine inhibition of glutamate uptake = 33 mM<br>K_m glutamate = 78 mM<br>K_m cystine = 45 mM	[12,14]
Glycine → Glycine	V_{max} = 47 h (0.01305 s), K_m = 67	[13]
Cysteine → Cysteine	V_{max} = 92 h (0.0255 s), K_m = 27	[13]

1. Ikeda Y, Fujii J, Taniguchi N. **Effects of substitutions of the conserved histidine residues in human gamma-glutamyl transpeptidase**. J Biochem. 1996 Jun;119(6):1166-70. doi: 10.1093/oxfordjournals.jbchem.a021363. PMID: 8827453.

2. Ikeda Y, Fujii J, Taniguchi N, Meister A. **Expression of an active glycosylated human gamma-glutamyl transpeptidase mutant that lacks a membrane anchor domain**. Proc Natl Acad Sci U S A. 1995 Jan 3;92(1):126-30. doi: 10.1073/pnas.92.1.126. PMID: 7816801; PMCID: PMC42830.

3.Kumar A, Tikoo S, Maity S, Sengupta S, Sengupta S, Kaur A, Bachhawat AK. **Mammalian proapoptotic factor ChaC1 and its homologues function as γ-glutamyl cyclotransferases acting specifically on glutathione**. EMBO Rep. 2012 Dec;13(12):1095-101. doi: 10.1038/embor.2012.156. Epub 2012 Oct 16. PMID: 23070364; PMCID: PMC3512401.

4. Kaur H, Kumar C, Junot C, Toledano MB, Bachhawat AK. **Dug1p Is a Cys-Gly peptidase of the gamma-glutamyl cycle of Saccharomyces cerevisiae and represents a novel family of Cys-Gly peptidases**. J Biol Chem. 2009 May 22;284(21):14493-502. doi: 10.1074/jbc.M808952200. Epub 2009 Apr 3. PMID: 19346245; PMCID: PMC2682898.

5. Weber P, Jäger M, Bangsow T, Knell G, Piechaczek K, Koch J, Wolf S. **Kinetic parameters and tissue distribution of 5-oxo-L-prolinase determined by a fluorimetric assay**. J Biochem Biophys Methods. 1999 Jan 13;38(1):71-82. doi: 10.1016/s0165-022x(98)00039-6. PMID: 10078874.

6. Jez JM, Cahoon RE, Chen S. **Arabidopsis thaliana glutamate-cysteine ligase: functional properties, kinetic mechanism, and regulation of activity**. J Biol Chem. 2004 Aug 6;279(32):33463-70. doi: 10.1074/jbc.M405127200. Epub 2004 Jun 4. PMID: 15180996.

7. Jez J. M., Cahoon R. E., Chen S. **Arabidopsis thaliana glutamate-cysteine ligase: functional properties, kinetic mechanism, and regulation of activity** //Journal of Biological Chemistry. – 2004. – Т. 279. – №. 32. – С. 33463-33470.

8. Jez J. M., Cahoon R. E. **Kinetic mechanism of glutathione synthetase from Arabidopsis thaliana** //Journal of Biological Chemistry. – 2004. – Т. 279. – №. 41. – С. 42726-42731.

9. Pader I, Sengupta R, Cebula M, Xu J, Lundberg JO, Holmgren A, Johansson K, Arnér ES. **Thioredoxin-related protein of 14 kDa is an efficient L-cystine reductase and S-denitrosylase**. Proc Natl Acad Sci U S A. 2014 May 13;111(19):6964-9. doi: 10.1073/pnas.1317320111. Epub 2014 Apr 28. PMID: 24778250; PMCID: PMC4024855.

10. Brandstaedter C. et al. **Kinetic characterization of wild-type and mutant human thioredoxin glutathione reductase defines its reaction and regulatory mechanisms** //The FEBS Journal. – 2018. – Т. 285. – №. 3. – С. 542-558.

11. Reed, M.C., Thomas, R.L., Pavisic, J. et al. **A mathematical model of glutathione metabolism**. Theor Biol Med Model 5, 8 (2008). https://doi.org/10.1186/1742-4682-5-8

12. Bridges RJ, Natale NR, Patel SA. **System xc^- cystine/glutamate antiporter: an update on molecular pharmacology and roles within the CNS**. Br J Pharmacol. 2012 Jan;165(1):20-34. doi: 10.1111/j.1476-5381.2011.01480.x. PMID: 21564084; PMCID: PMC3252963.

13. Raftos JE, Whillier S, Kuchel PW. **Glutathione synthesis and turnover in the human erythrocyte: alignment of a model based on detailed enzyme kinetics with experimental data**. J Biol Chem. 2010 Jul 30;285(31):23557-67. doi: 10.1074/jbc.M109.067017. Epub 2010 May 24. PMID: 20498365; PMCID: PMC2911318.

14. Lewerenz J, Hewett SJ, Huang Y, Lambros M, Gout PW, Kalivas PW, Massie A, Smolders I, Methner A, Pergande M, Smith SB, Ganapathy V, Maher P. **The cystine/glutamate antiporter system x(c)(-) in health and disease: from molecular mechanisms to novel therapeutic opportunities**. Antioxid Redox Signal. 2013 Feb 10;18(5):522-55. doi: 10.1089/ars.2011.4391. Epub 2012 Aug 3. PMID: 22667998; PMCID: PMC3545354.
